# Author Correction: Asymmetric sheath coordination controls flagellar architecture and function in *Leptospira* spirochete

**DOI:** 10.1038/s44318-026-00859-0

**Published:** 2026-07-16

**Authors:** Akihiro Kawamoto, Toshiki Kuribayashi, Masatomo Morita, Shuichi Nakamura, Nobuo Koizumi

**Affiliations:** 1 Institute for Protein Research, The University of Osaka, 3-2 Yamadaoka, Suita, Osaka 565-0871 Japan; 2 Department of Applied Physics, Graduate School of Engineering, Tohoku University, 6-6-05 Aoba, Aoba, Sendai, Miyagi 980-8579 Japan; 3 Department of Bacteriology I, National Institute of Infectious Diseases, Japan Institute for Health Security, 1-23-1 Toyama, Shinjuku, Tokyo, 162-8640 Japan

## Abstract

**Figure:**
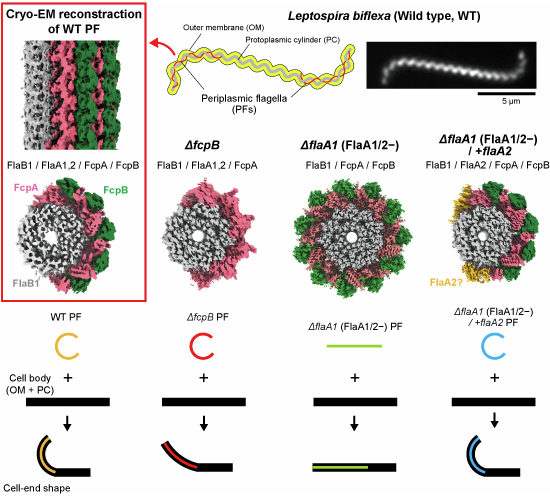

## Graphical abstract

**Figure:**
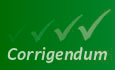

**Correction to:** *The EMBO Journal* (2026) 45, 2882–2904. 10.1038/s44318-026-00731-1 | Published online 17 March 2026

**Manuscript Synopsis image is corrected**.

**Figure:**
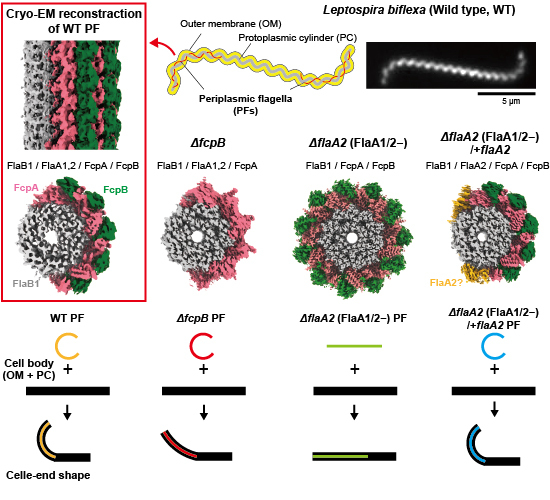

In the originally published manuscript, an error was introduced in the Synopsis figure.

The label “ΔflaA1” was incorrectly shown and should read “ΔflaA2”.

This has now been corrected in the online version of the Synopsis figure.

The authors apologise for this oversight. This correction does not affect the results or conclusions of the article.

