# Supplementary material for: Asymmetric sheath coordination controls flagellar architecture and function in Leptospira spirochete
Source: EMBO J. 2026 Mar 17;45(9):2882–904. doi: 10.1038/s44318-026-00731-1 (PMC13144727; doi:10.1038/s44318-026-00731-1)
Supplement: Supplementary file 1 — Appendix [file 44318_2026_731_MOESM1_ESM.pdf]

Appendix for

"Asymmetric sheath coordination controls flagellar architecture and function in Leptospira spirochete"

Table of contents

Appendix Table S1 2

Appendix Table S2 3

Appendix Table S3 5

Appendix Figure S1 8

Appendix Figure S2 9

Appendix Figure S3 10

Appendix Figure S4 11

Appendix Figure S5 13

Appendix Figure S6 14

Appendix Figure S7 16

Appendix Figure S8 18

Appendix Figure S9 19

Appendix Figure S10 20

Appendix Figure S11 21

Appendix Figure S12 23

Appendix Figure S13 25

Appendix Figure S14 26

Appendix Table S1. Peptides identified by MALDI-TOF mass spectrometry and matched to FcpB of *Leptospira biflexa*.

Peptide sequence	Start-End	Score	Mr (calc)	Observed m/z	ppm	Modification(s)	Unique
VISVIADVAPNGEPAHNDK	171-189	104	1944.9905	1945.9819	–8.16	—	U
MELFFEGSNADPDPR*	131-146	86	1837.7941	1838.7906	–5.89	—	U
TYIYQNNFLLEDK	158-170	76	1659.8144	1660.8128	–5.34	—	
IFDYNDRDSNK	252-262	59	1385.6211	1386.6167	–8.43	—	
IELFYQHDDYPVWGTPETPSEK	190-211	56	2650.2227	2651.2127	–6.55	—	
YILSNVENTK	216-225	55	1179.6135	1180.5985	–18.90	—	
ELDDNISEVNKR#	47-58	29	1430.7001	1431.6970	–7.28	—	

The position of each peptide within the protein sequence is shown (Start–End), along with the observed mass-to-charge ratio (m/z), calculated molecular weight (Mr), mass error (ppm), Mascot score, and any detected modifications. "Unique" indicates peptides uniquely matching the target protein.

*The correct theoretical sequence includes an additional asparagine: "MELFFEGSNNADPDPR".

#Although this peptide corresponds to the N-terminus, it was identified with a relatively low Mascot score and high expect value, and thus should be interpreted with caution.

Appendix Table S2. Primer sequences used in this study.

Target	Primer sequence (5´→ 3´)
	Forward
fcpB upstream region	GCGTCGACGGTATCGGAAGGGATGGTTGGAACTGA
fcpB downstream region	ACAGGTGCTTACTTTCCCCATCGGCACTTGGTTTTC
flaA1 upstream region	CTGGCCGGCGTCGACGGTATCCTGCATCAGCACGAACATAAA
flaA1 downstream region	CAATAACAGGTGCTTACTTTAGCTCTGGTGACTTCATACTC
flaA2 upstream region	CTGGCCGGCGTCGACGGTATCCTGGAGCTTCAATTCCATAC
flaA2 downstream region	CAATAACAGGTGCTTACTTTGGGTATAATTCCACACGATCC
flaA2	TATCGATACCGTCGAGCCAGCGCAAGAGACA
Kanamycin resistance cassette	GGAACCTCTTACGTGCCGATC
pNKLbKmR (pCjSpLe94-derived)	TCTAGGCACCAATAACTGCCCATCAGAGTATGGACAGTTGC

Appendix Table S2. (continued)

Reverse
TCCTTGAAGCTCGGGAGCGTTGTGAAGCTGCAGAC
ATATCAAGCTTATCGCGGGTTGTTTGAAGCGACTAG
AATCTTCCTTGAAGCTCGGGCGTTCGTACATCGATGCAGAC
GAATTCGATATCAAGCTTATCTGGATCGTGTGGAATTATACC
AATCTTCCTTGAAGCTCGGGTCCGCATGAATACATCATCAAAGG
GAATTCGATATCAAGCTTATCGGTATGCCGATTTGCATGA
CTCTGCGAGGCTGGCCGGCGTTACCAATTGTCCTTAATTTCAG
GGCAGTTATTGGTGCCTAGAAATA
ATCGGCACGTAAGAGGTTCCATCGGCTCCGTCGATACTAT

Appendix Table S3. Cryo-EM data and refinement statistics

Strain name	Wild type		*ΔfcpB*_CL15
Filament	core (FlaB2)	sheathed (FlaB1, FcpA, FcpB)	core (FlaB2)
Curved	+	+	-
PDB	9X7K	9X80	9LS1
EMD	EMD-66641	EMD-66649	EMD-63350
Microscope	Titan Krios		Titan Krios
Magnification	59,000		59,000
Voltage (kV)	300		300
Detector	Falcon3 (Counting)		Falcon3 (Counting)
Electron exposure (e-/A2)	50 (109 frames)		50 (108 frames)
Defocus range (μm)	-0.5 to -2		-0.5 to -2
Pixel size (Å)	1.13		1.13
Initial particles images (no.)	857,159		1,171,169
Final particle images (no.)	77,007	457,393	350,712
Helical twist (deg)	-	-	65.41
Helical rise (Å)	-	-	4.793
Map resolution (Å)	4.35	3.24	2.37
FSC threshold	0.143	0.143	0.143
Map resolution range (Å)	2.41-7.96	2.47-5.50	2.26-3.60
Map sharpening B factor (Å2)	-47.2	-75.5	-52.46
Refinement			
Initial model used (PDB code)	9LRZ	9LRY	9LRZ
d model	4.4	4.4	2.4
d FSC model (0/0.143/0.5)	4.1/4.4/7.4	3.2/3.3/4.1	2.2/2.2/2.5
Model composition			
Non-hydrogen atoms	23,903	48,078	2,171
Protein residues	3,080	5,961	280
Ligands	0	0	0
R.m.s deviations			
Bond lengths	0.003	0.002	0.005
Bond angles	0.624	0.371	0.515
Validation			
MolProbity score	1.78	1.36	1.39
Clash score	12.98	4.68	3.46
Poor rotamers	-	1.42	2.14
Ramachandran plot			
Favored	97.12	98.32	99.28
Allowed	2.88	1.68	0.72
Disallowed	0	0	0

Appendix Table S3. (continued)

	ΔflaA2		*flaA2*-complemented, *ΔflaA2*
sheathed (FlaB1, FcpA)	core (FlaB2)	sheathed (FlaB1, FcpA, FcpB)	core (FlaB2)
-	-	-	+
9LS0	9LRZ	9LRY	9X7L
EMD-63349	EMD-63348	EMD-63347	EMD-66642
	Titan Krios		Titan Krios
	59,000		81,000
	300		300
	Falcon3 (Counting)		K3 (Counting)
	50 (109 frames)		40 (40 frames)
	-0.5 to -2		-0.5 to -1
	1.13		0.87→ 1.16
	1,953,561		1,953,561
203,738	273,749	138,670	457,214
65.44	65.41	65.43	-
4.82	4.79	4.84	-
2.37	2.31	2.32	3.31
0.143	0.143	0.143	0.143
2.26-4.4	2.26-3.64	2.26-3.84	2.53-5.60
-50.5914	-46.1808	-45.5861	-36.1
9LRY	-	-	9LRZ
2.4	2.4	2.3	3.5
2.1/2.2/2.5	2.2/2.2/2.4	2.2/2.2/2.4	3.1/3.3/3.7
4,280	2,171	6,208	23,903
525	280	761	3,080
0	0	0	0
0.003	0.004	0.002	0.003
0.501	0.48	0.445	0.57
1.46	1.03	1.27	1.92
3.51	1.84	3.88	9.11
2.6	1.28	1.33	3.77
99.23	99.28	98.54	98.14
0.77	0.72	1.46	1.86
0	0	0	0

Appendix Table S3. (continued)

	*ΔfcpB*_CL13	
sheathed (FlaB1, FcpA, FcpB)	core (FlaB2)	sheathed (FlaB1, FcpA)
+	+	+
9X7S	9X7M	9X7V
EMD-66646	EMD-66643	EMD-66647
	Titan Krios	
	81,000	
	300	
	K3 (Counting)	
	40 (40 frames)	
	-0.5 to -1	
	0.87→ 1.16	
	8,206,013	
2,667,344	832,792	3,125,674
-	-	-
-	-	-
2.83	3.05	2.7
0.143	0.143	0.143
2.60-3.79	2.70-4.86	2.60-3.70
-69.1	-40.8	-59.7
9LRY	9LRZ	9LRY
3	3.2	2.8
2.7/2.8/3.3	2.9/3.03/3.4	2.6/2.7/16.8
50,274	23,903	39,198
6,218	3,080	4,868
0	0	0
0.002	0.003	0.003
0.4	0.506	0.444
1.5	1.85	1.28
4.85	7.79	5.25
2.11	3.61	0.95
98.18	98.2	98.65
1.82	1.8	1.35
0	0	0

A

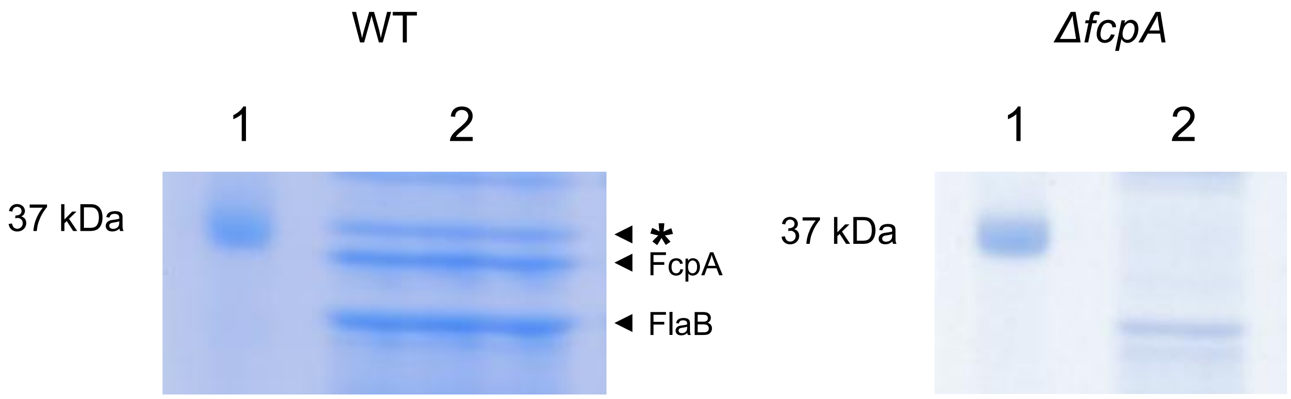

B

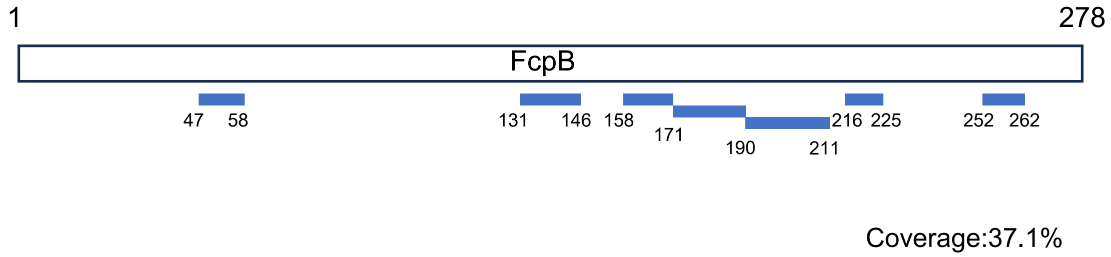

Appendix Figure S1. Identification of FcpB as a protein absent from periplasmic flagella (PFs) purified from *ΔfcpA* mutant.
(A) Coomassie Brilliant Blue staining of purified PFs from wild type (WT) and *ΔfcpA* mutant of *L. biflexa*, separated by 10% SDS-PAGE. The band marked with an asterisk corresponds to FcpB. PFs were prepared in two independent experiments, and each preparation was analyzed by SDS-PAGE; a representative gel is shown. Lanes 1, molecular weight marker; 2, purified PFs.
(B) Schematic representation of peptide fragments of FcpB identified by MALDI-TOF-MS/MS, indicated in blue.

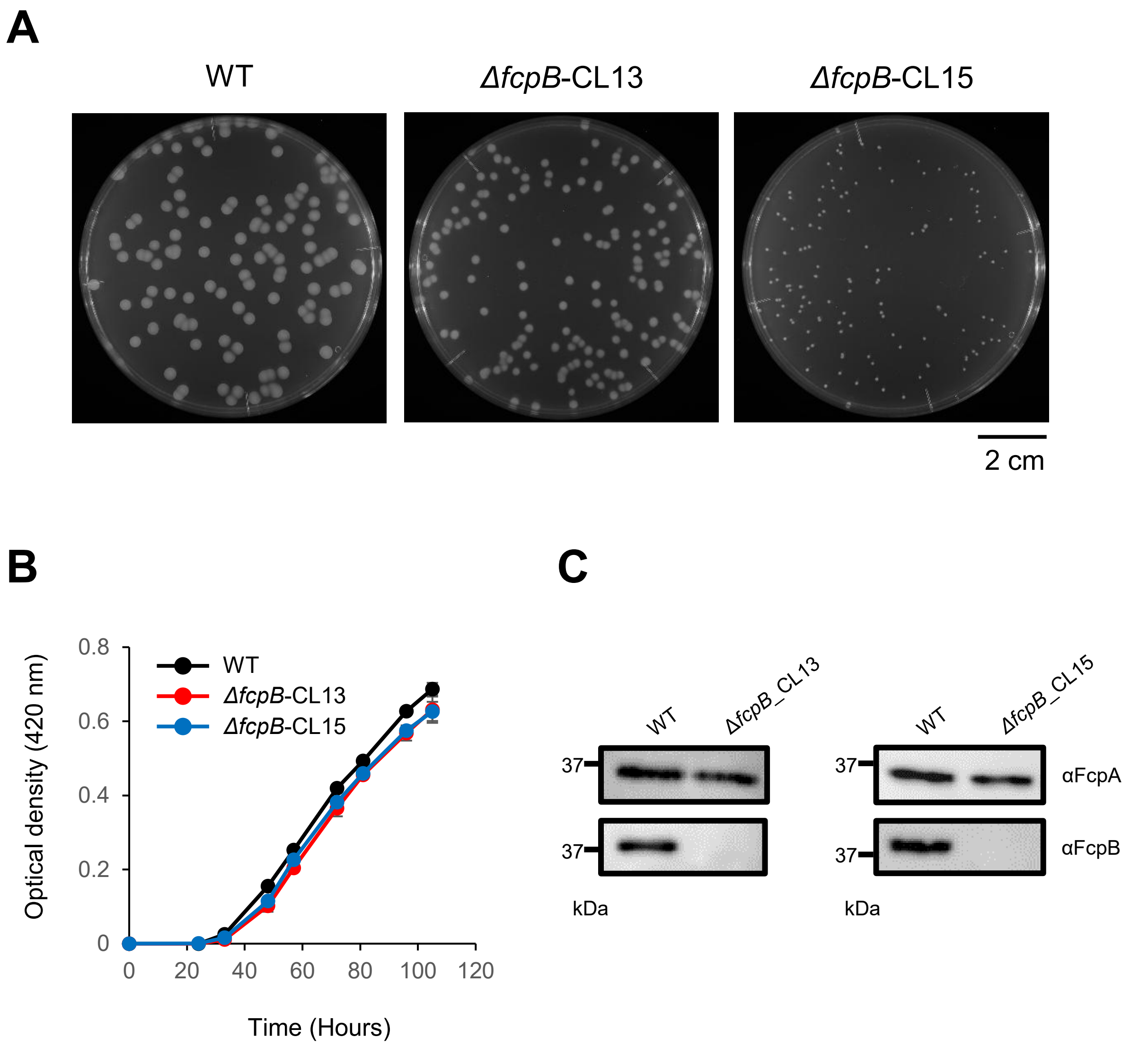

Appendix Fig. S2. Phenotypic differences between *ΔfcpB* mutants *ΔfcpB*-CL13 and *ΔfcpB*-CL15.

A Colony morphology on 0.4% soft agar plates after 7 days of incubation at 30℃. The assay was performed twice independently, and representative images are shown. The WT colony image is reused from Fig. 4A. WT, wild type.

B Growth of *L. biflexa* WT (black), *ΔfcpB*_CL13 (red), and *ΔfcpB*_CL15 (blue). Optical density (OD) at 420 nm was measured at 0, 24, 33, 48, 51, 72, 81, 96, and 105 h after inoculation. Measurements were terminated at 105 h (day 4), as cell sedimentation occurred after day 5. For each independent experiment, OD measurements were performed in triplicate, and the triplicate values were averaged to obtain a single data point. Data points represent the mean of three independent experiments, and error bars indicate the standard deviation.

C Immunoblotting of purified periplasmic flagella (PFs) from WT, *ΔfcpB*-CL13, and *ΔfcpB*-CL15 strains using anti-FcpA and anti-FcpB antisera. PFs were prepared in two independent experiments, and each preparation was analyzed by immunoblotting; a representative blot is shown. The immunoblot images probed with anti-FcpA and anti-FcpB are reused from Fig. 4C.

A

```
              90                  95                 100                 105                 110                 115
WT            D   G   G   D   K   I   E   K   N   H   I   L   G   V   K   T   H   F   A   A   K   G   L   D   R   V   E   L
              GAT GGT GGG GAC AAA ATC GAA AAA AAC CAT ATT TTA GGA GTC AAA ACA CAT TTT GCT GCT AAA GGG CTG GAT CGT GTG GAA TTA
              GAT GGT GGG GGA CAA AAT CGA AAA AAA CCA TAT TTT AGG AGT CAA AAC ACA TTT TGC TGC TAA AGG GCT GGA TCG TGT GGA ATT
ΔfcpB_CL15    D   G   G   G   Q   N   R   K   K   P   Y   F   R   S   Q   N   T   F   C   C   *
```

B

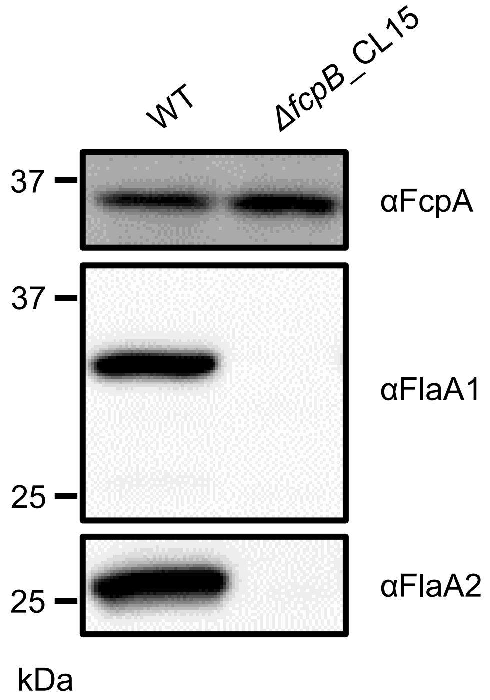

Appendix Fig. S3. Amino acid and nucleotide sequences of the *flaA2* gene and expression of FlaA2 in wild type (WT) and *ΔfcpB* mutant (*ΔfcpB*_CL15).
A A one-nucleotide insertion in the *flaA2* gene of *ΔfcpB*_CL15 is indicated in red.
B Immunoblotting of whole-cell lysates from WT and *ΔfcpB*_CL15 strains using anti-FcpA, anti-FlaA1, and anti-FlaA2 antisera. Whole-cell lysates were prepared in two independent experiments, and each preparation was analyzed by immunoblotting; a representative blot is shown.

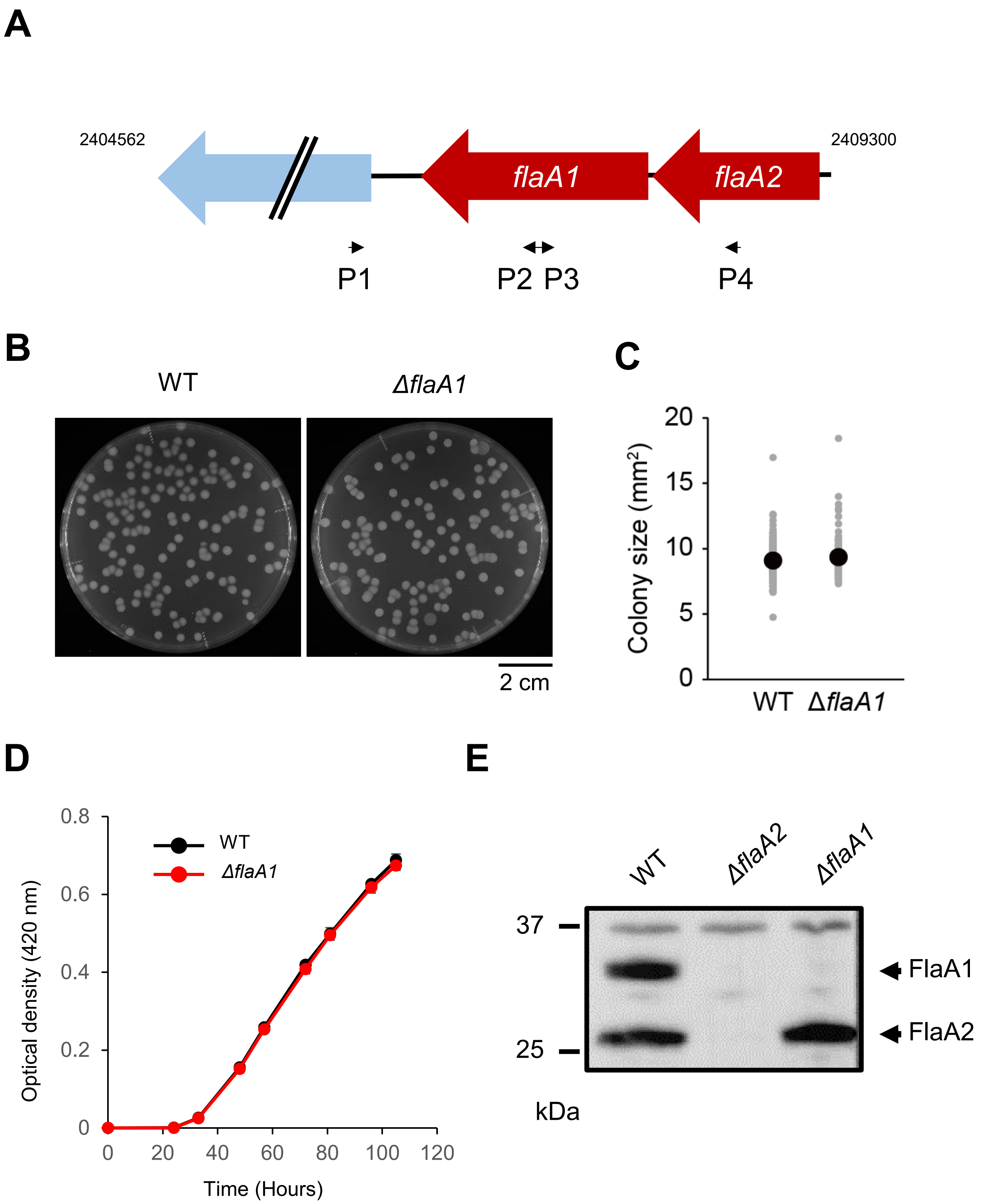

Appendix Fig. S4. Colony size and expression of FlaA proteins in *ΔflaA1* mutant.
A Schematic diagram of the genomic region (positions 2404562 to 2409300) of *L. biflexa* serovar Patoc strain Patoc I (Paris) (GenBank accession no. CP000786.1), showing the relative positions and orientations of *flaA2* and *flaA1*. The locations of primers used for constructing the *flaA1* deletion mutant (P1: 2407227–2407248, P2: 2408027–2408047, P3: 2408048–2408068, P4: 2408897–2408918) are indicated by black arrows. The gene shown in blue is partially truncated for simplicity, as denoted by the double slash (//).

B Colony morphology on 0.4% soft agar plates after 7 days of incubation at 30℃. The assay was performed twice independently, and representative images are shown. WT, wild-type. The WT colony image is reused from Fig. 2A.
C Quantification of colony size of WT and *ΔflaA1* strains. All individual data points are plotted, with mean values indicated by black circles. Data were collected from 131 colonies for WT and 104 colonies for *ΔflaA1*, obtained from two independent biological replicates. Statistical significance was assessed using a two-tailed Student's t-test, assuming unequal variances ($P = 0.21$).
D Growth of *L. biflexa* WT (black) and *ΔflaA1* (red). Optical density (OD) at 420 nm was measured at 0, 24, 33, 48, 51, 72, 81, 96, and 105 h after inoculation. Measurements were terminated at 105 h (day 4), as cell sedimentation occurred after day 5. For each independent experiment, OD measurements were performed in triplicate, and the triplicate values were averaged to obtain a single data point. Data points represent the mean of three independent experiments, and error bars indicate the standard deviation.
E Immunoblotting of whole-cell lysates from WT, *ΔflaA2*, and *ΔflaA1* strains using anti-FlaA1 and anti-FlaA2 antisera. Whole-cell lysates were prepared in two independent experiments, and each preparation was analyzed by immunoblotting; a representative blot is shown.

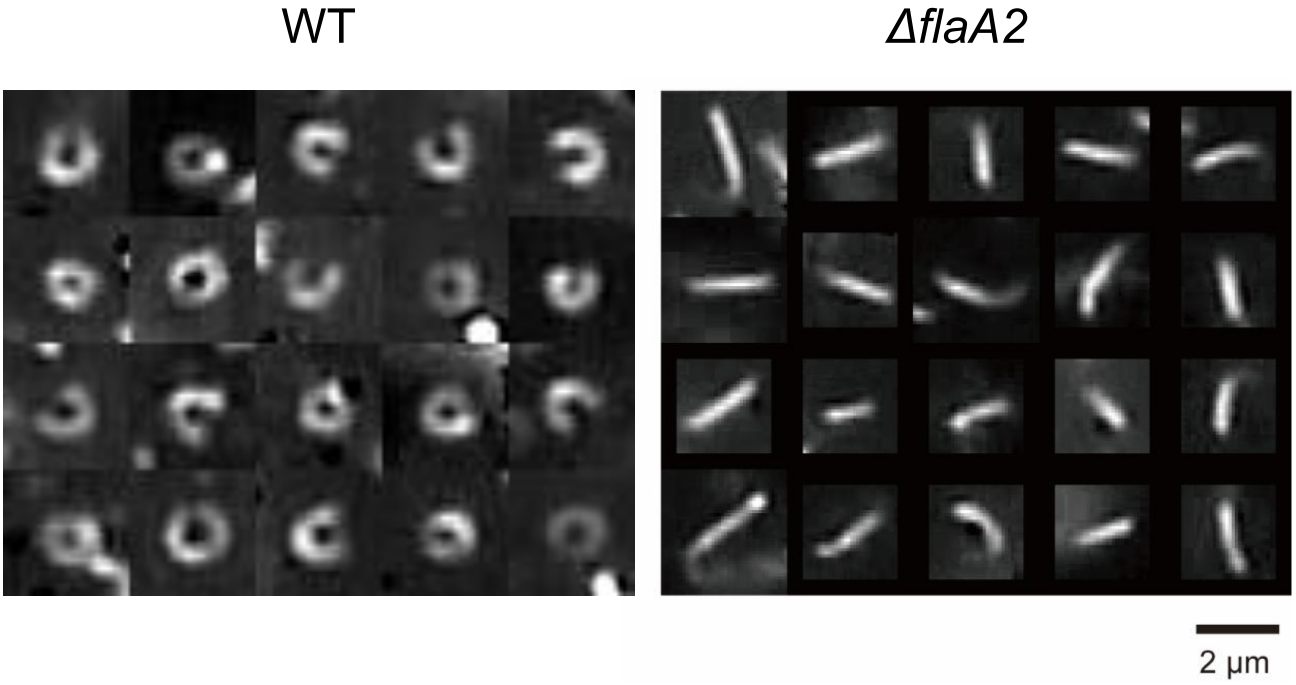

Appendix Fig. S5. Dark-field microscopy images of periplasmic flagella (PFs) isolated from wild-type (WT) and *ΔflaA2* strains.
WT PFs show curved morphology, whereas ΔflaA2 PFs are straight.

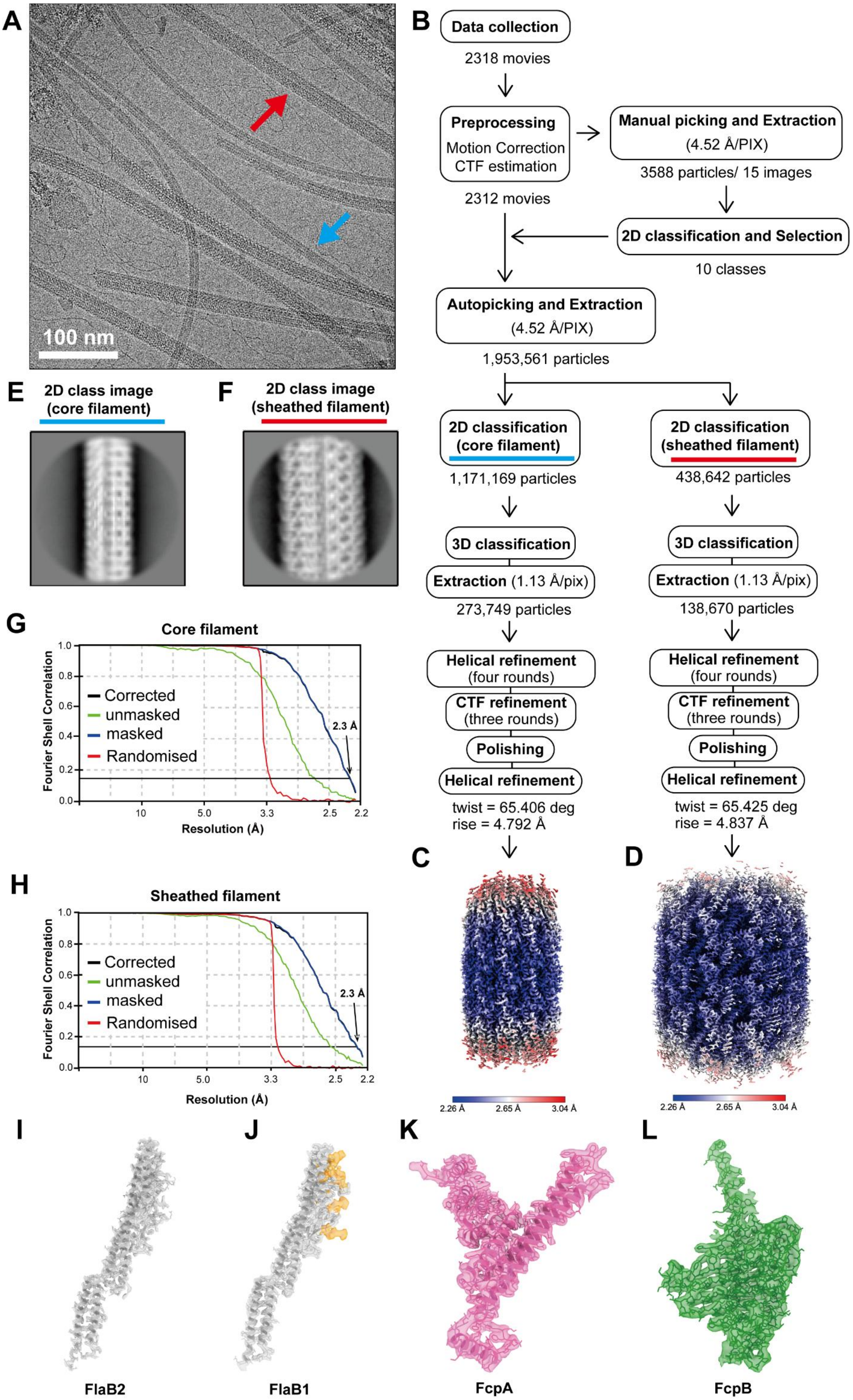
A
100 nm
B
Data collection
2318 movies
Preprocessing
Motion Correction
CTF estimation
2312 movies
Manual picking and Extraction
(4.52 Å/PIX)
3588 particles/ 15 images
2D classification and Selection
10 classes
Autopicking and Extraction
(4.52 Å/PIX)
1,953,561 particles
2D classification
(core filament)
1,171,169 particles
2D classification
(sheathed filament)
438,642 particles
3D classification
Extraction (1.13 Å/pix)
273,749 particles
3D classification
Extraction (1.13 Å/pix)
138,670 particles
Helical refinement
(four rounds)
CTF refinement
(three rounds)
Polishing
Helical refinement
twist = 65.406 deg
rise = 4.792 Å
Helical refinement
(four rounds)
CTF refinement
(three rounds)
Polishing
Helical refinement
twist = 65.425 deg
rise = 4.837 Å
E
2D class image
(core filament)
F
2D class image
(sheathed filament)
G
Core filament
Fourier Shell Correlation
Corrected
unmasked
masked
Randomised
2.3 Å
Resolution (Å)
H
Sheathed filament
Fourier Shell Correlation
Corrected
unmasked
masked
Randomised
2.3 Å
Resolution (Å)
C
D
2.26 Å
2.65 Å
3.04 Å
I
J
K
L
FlaB2
FlaB1
FcpA
FcpB

Appendix Fig. S6. Summary of Cryo-EM data acquisition and image processing of the periplasmic flagella (PFs) from *ΔflaA2* strain.
A Cryo-EM image of purified PFs. Blue and red arrows indicate the unsheathed core filament and the sheathed filament, respectively.
B Data processing workflow.
C Reconstructed image of the unsheathed core filament.
D Reconstructed image of the sheathed filament.
E 2D class image of the unsheathed core filament.
F 2D class image of the sheathed filament.
G Fourier shell correlation (FSC) analysis of the reconstructed unsheathed core filament, showing a global resolution of 2.3 Å.
H FSC analysis of the reconstructed sheathed filament, showing a global resolution of 2.3 Å.
I–L Fitted atomic models and corresponding density maps of FlaB2 (**I**), FlaB1 (**J**), FcpA (**K**), and FcpB (**L**).

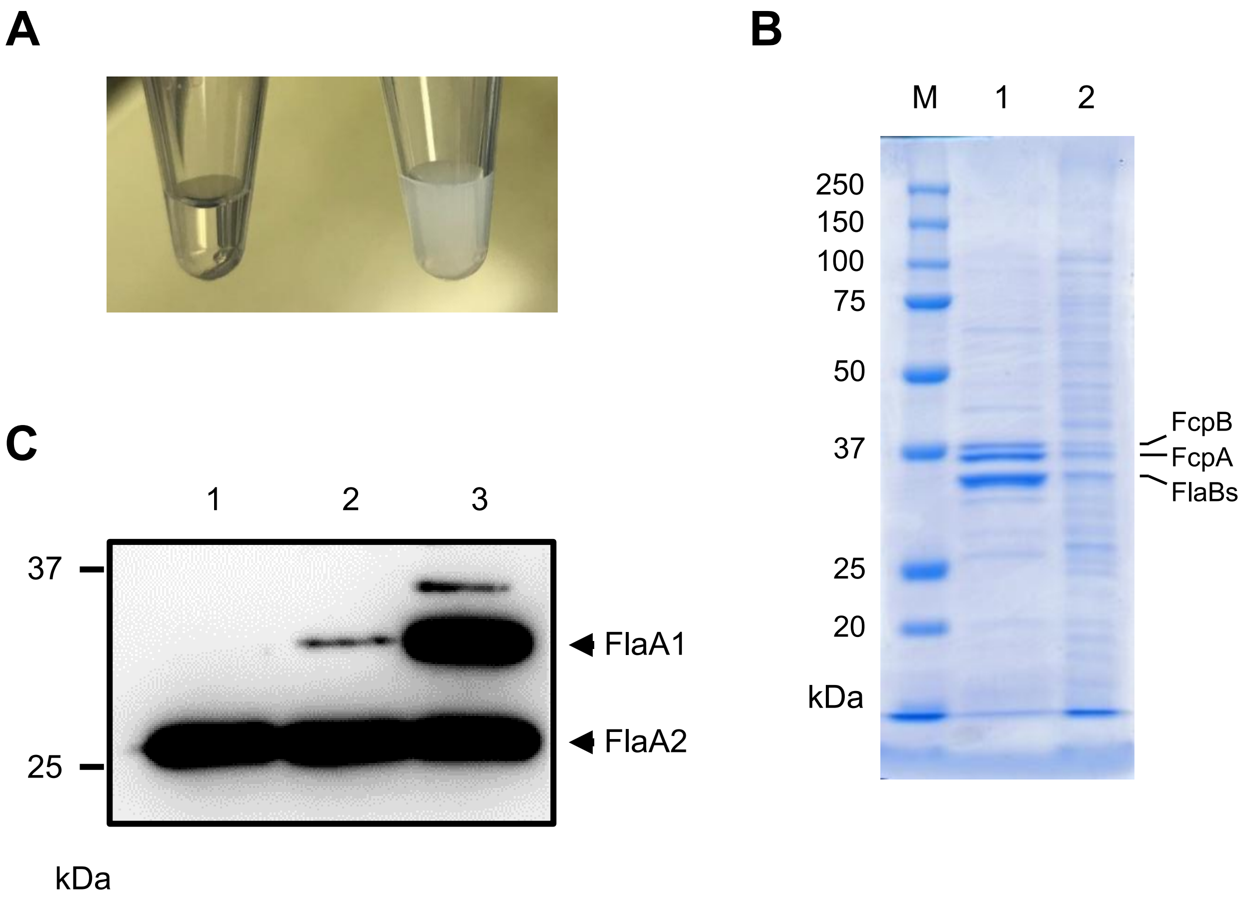

Appendix Fig. S7. Comparison of periplasmic flagella (PFs) purified by different methods.

A Photographs of purified PF fractions. PFs were prepared in two independent experiments, and a representative image is shown. Left, PF fraction purified by the method described in this study; right, PF fraction purified by the method described in Wunder et al. (2016).

B Coomassie Brilliant Blue staining of purified PFs (1.5 μg) on 10% SDS-PAGE. PFs were prepared in two independent experiments, and each preparation was analyzed by SDS-PAGE; a representative gel is shown. Lanes: M, molecular weight marker; 1, PF fraction purified by the method described in this study; 2, PF fraction purified by the method described in Wunder et al. (2016).

C Immunoblotting of purified PFs (1.5 μg) using anti-FlaA1 and anti-FlaA2 antisera. PFs were prepared in two independent experiments, and each preparation was analyzed by immunoblotting; a representative blot is shown. Lanes: 1, PF fraction purified by the method described in this study; 2, PF fraction purified by the method described in Wunder et al. (2016); 3, whole-cell lysate.

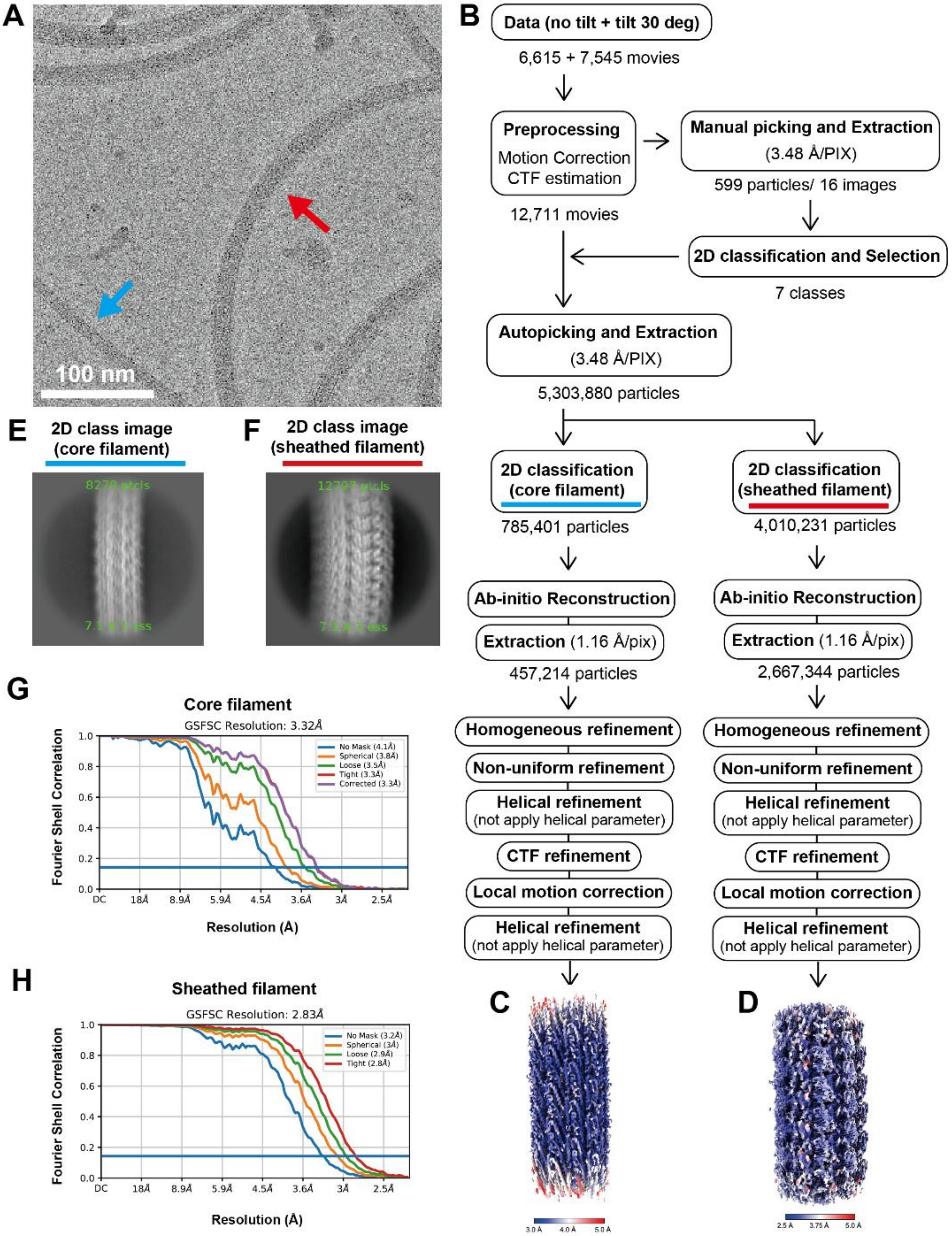

Appendix Fig. S8. Summary of Cryo-EM data acquisition and image processing of the periplasmic flagella (PFs) from *flaA2*-complemented, *ΔflaA2* strain.

A Cryo-EM image of purified PFs. Blue and red arrows indicate the unsheathed core filament and the sheathed filament, respectively.

B Data processing workflow.

C Reconstructed image of the unsheathed core filament.
D Reconstructed image of the sheathed filament.
E 2D class image of the unsheathed core filament.
F 2D class image of the sheathed filament.
G Fourier shell correlation (FSC) analysis of the reconstructed unsheathed core filament, showing a global resolution of 3.32 Å.
H FSC analysis of the reconstructed sheathed filament, showing a global resolution of 2.83 Å.

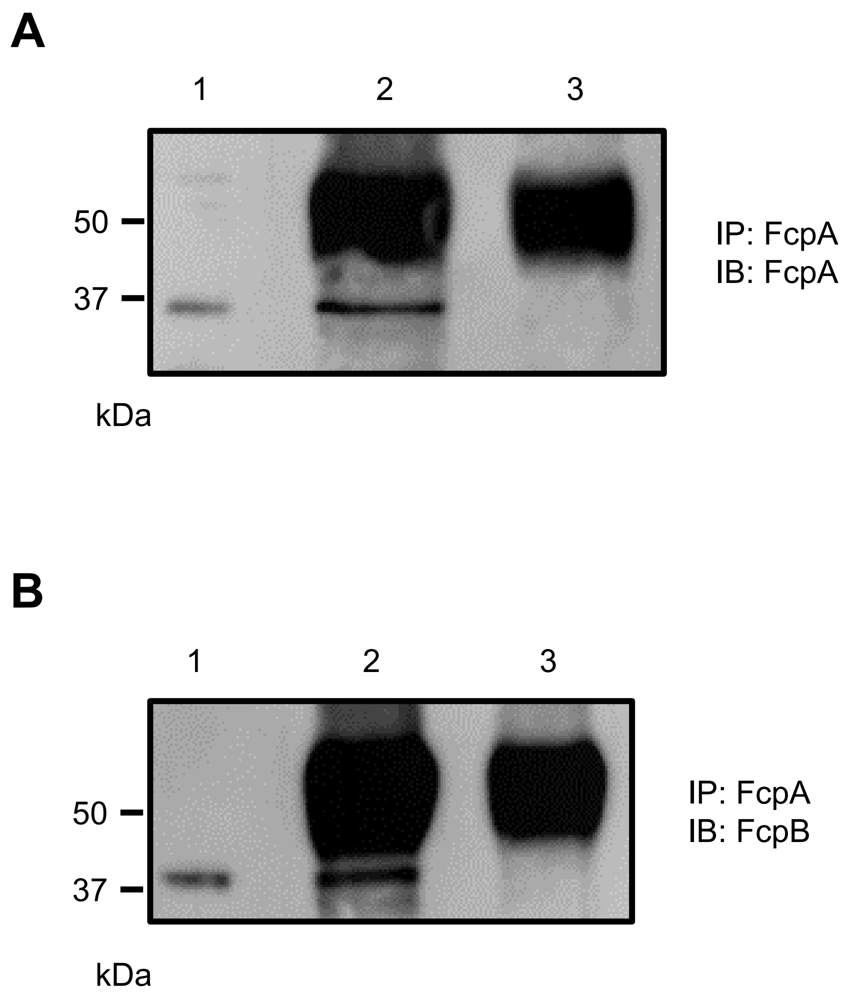

Appendix Fig S9. Immunoprecipitation of FcpB with anti-FcpA. Lysates from wild-type strain were immunoprecipitated using anti-FcpA antiserum and then probed with antisera against FcpA (**A**) and FcpB (**B**). Whole-cell lysates were prepared in two independent experiments, and each preparation was analyzed by immunoprecipitation followed by immunoblotting; representative blots are shown. *IP*, immunoprecipitation; *IB*, immunoblotting. Lanes: 1, whole cell lysates; 2, immunoprecipitates with anti-FcpA antiserum; 3, immunoprecipitates with preimmune serum.

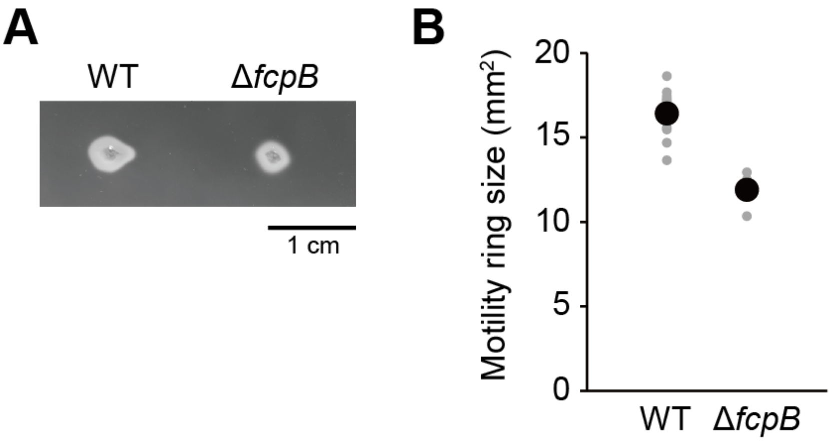

Appendix Fig S10. Soft-agar motility assay of wild-type (WT) and *ΔfcpB* (*ΔfcpB*_CL13) strains.
A Representative growth of WT and *ΔfcpB*_CL13 strains inoculated into 0.4% soft agar plate after 5 days of incubation at 30℃. The assay was performed twice independently, with four plates in each experiment.
B Quantification of motility ring size of WT and *ΔfcpB*_CL13 strains. All individual data points are plotted, with mean values indicated by black circles. Data were collected from eight plates, obtained from two independent biological replicates. Statistical significance was assessed using a two-tailed Student's t-test, assuming unequal variances (P = 3.50 × 10^{-10}).

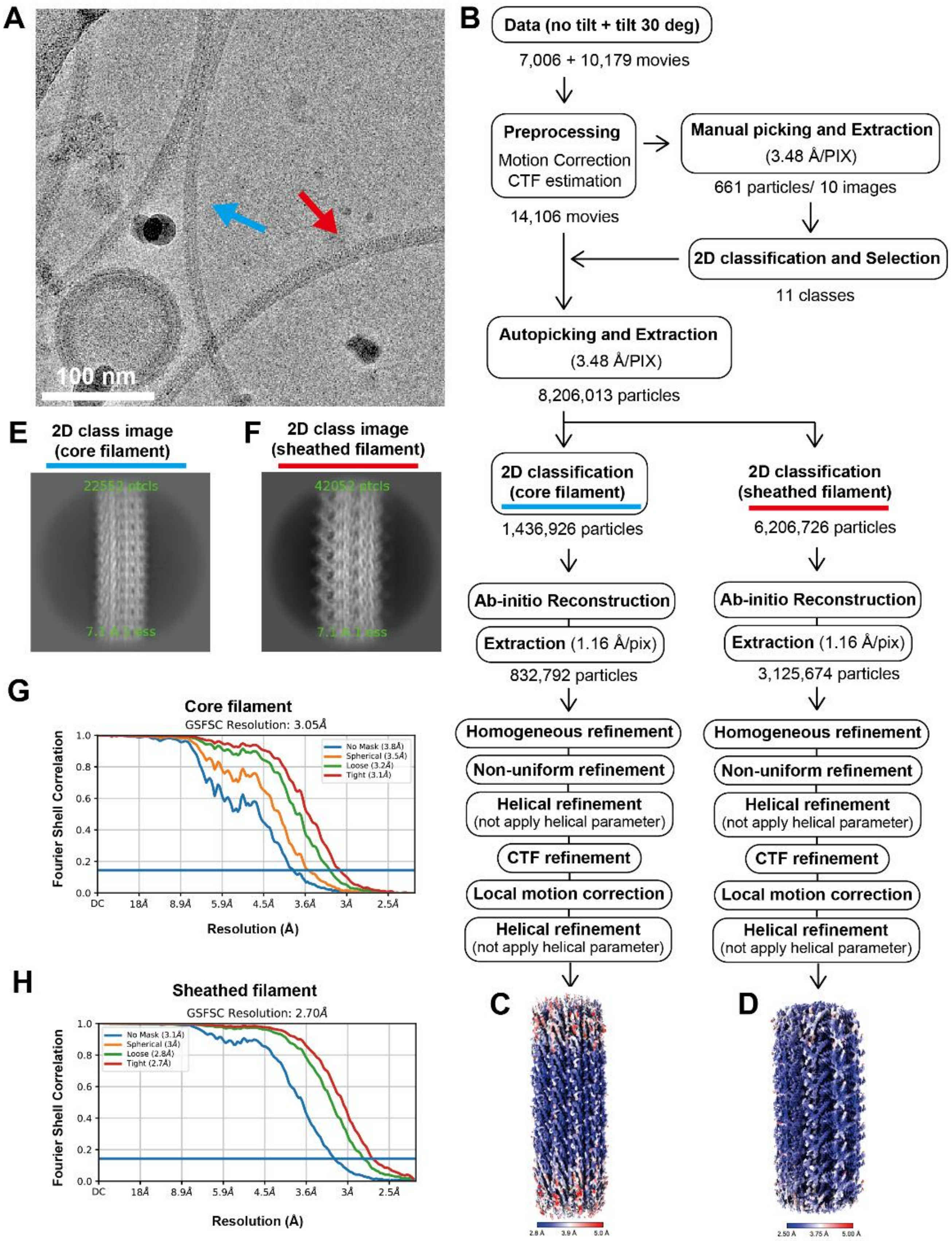

Appendix Fig. 11. Summary of Cryo-EM data acquisition and image processing of the periplasmic flagella (PFs) from *ΔfcpB*_CL13 strain.

A Cryo-EM image of purified PFs. Blue and red arrows indicate the unsheathed core filament and the sheathed filament, respectively.

B Data processing workflow.

C Reconstructed image of the unsheathed core filament.
D Reconstructed image of the sheathed filament.
E 2D class image of the unsheathed core filament.
F 2D class image of the sheathed filament.
G Fourier shell correlation (FSC) analysis of the reconstructed unsheathed core filament, showing a global resolution of 3.05 Å. (H) FSC analysis of the reconstructed sheathed filament, showing a global resolution of 2.70 Å.

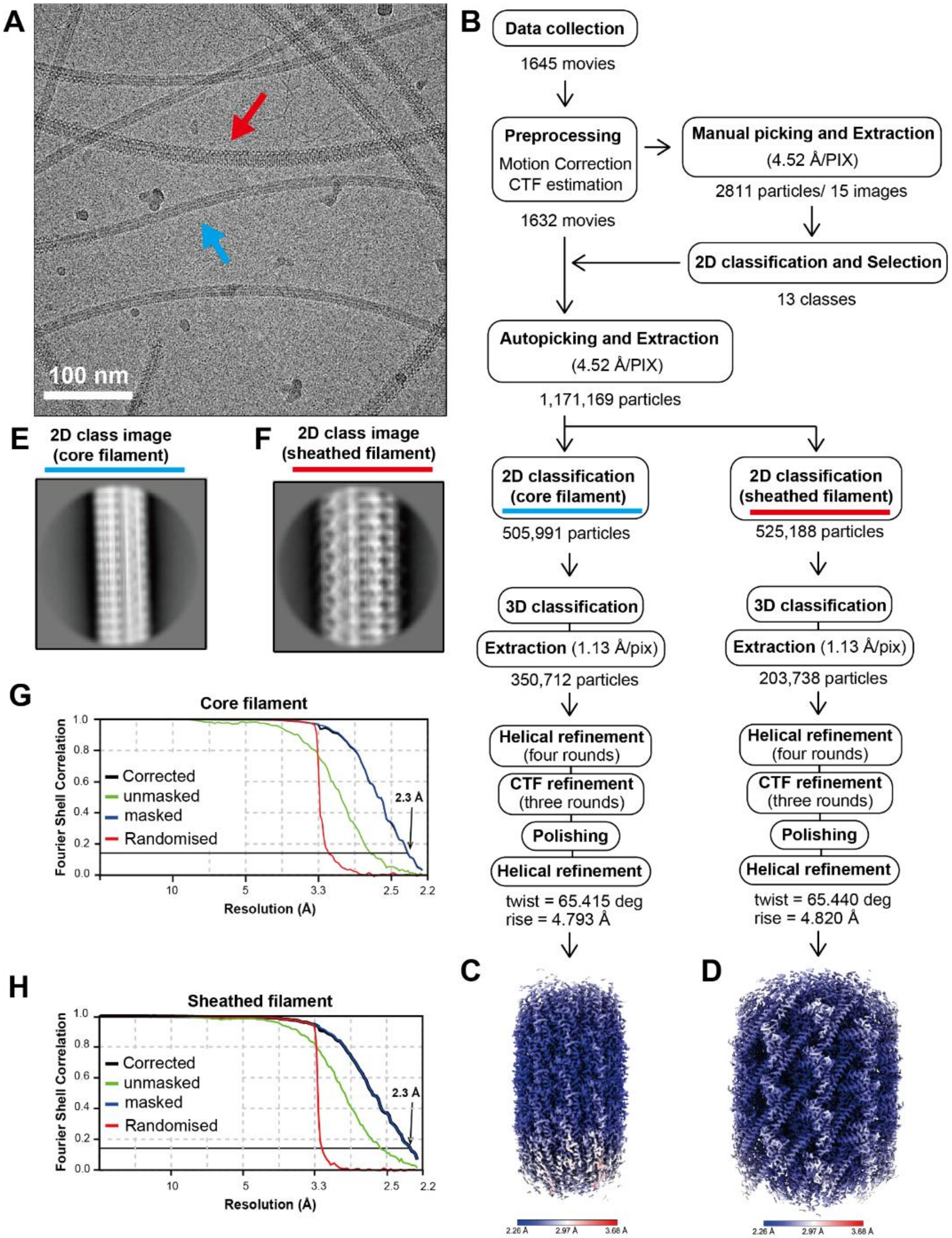

Appendix Fig. S12. Summary of Cryo-EM data acquisition and image processing of the periplasmic flagella (PFs) from *ΔfcpB*_CL15 strain.

A Cryo-EM image of purified PFs. Blue and red arrows indicate the unsheathed core filamaent and the sheathed filament, respectively.

B Data processing workflow.

C Reconstructed image of the unsheathed core filament.
D Reconstructed image of the sheathed filament.
E 2D class image of the unsheathed core filament.
F 2D class image of the sheathed filament.
G Fourier shell correlation (FSC) analysis of the reconstructed unsheathed core filament, showing a global resolution of 2.3 Å.
H FSC analysis of the reconstructed sheathed filament, showing a global resolution of 2.3 Å.

```
FlaB1   1:MIINHNVSAIFAHRTLKSNDANLSKDIEKLSSGMRINKAGDDASGLAVSEKMRTQIAGLR 60
FlaB2   1:MIINHNLAAINSHRVLKFQNEEVSKNMEKLSSGMRINRAGDDASGLAVSEKMRTQVNGLR 60
          ******  **  ** **      **  ********** *****************  ***

FlaB1  61:RAEQNTEDGMSLIQTAEGYLQETHEIVQRVRVLAVQAANGIYSEEDRQQIQVEVSQLVDE 120
FlaB2  61:QAERNTEDGMSLIQTTEGFLQESNDIIQRIRTLAIQSSNGIYTEEDRQMIQVEVSQLIDE 120
           ** *********** ** ***   * ** * ** *  **** ***** ******** **

FlaB1 121:IDRIASQAEFNKMKLLTGAFARLNPTASMWFHIGANMHQRERVYIETMNTAALGLRNPTV 180
FlaB2 121:VDRIASQAEFNKMNLLQGDFARGSRATSMWFHIGPNMHQRERVFIATMTARSLNLKGQSG 180
           ************ ** * ***     ******* ******** * **    * *

FlaB1 181:LTFISLSTAGKANSVIGLCDDALRVISKQRADLGAYYNRMEHAAKGLMNAYENTQASESR 240
FlaB2 181:-ELLSLSTADKSNDAIGTLDAALTRISKQRANLGAYFNRLEHAAKGLMNAYENTQASESR 239
              ***** * *  **  * **  ****** **** ** ********************

FlaB1 241:IRDTDMAEQMTSFTRYQILTQAATSMLAQANMKSQSVMRLLQ                   282
FlaB2 240:IRDADMAEETVAFTKNQILVQSGTAMLAQANVRPQGVLSLLR                   281
          *** ****    **  *** *  * ******   * *  **
```

Appendix Fig. S13. Comparison of the amino acid sequences of FlaB1 and FlaB2. Glycosylated amino acids in FlaB1 are marked in red.

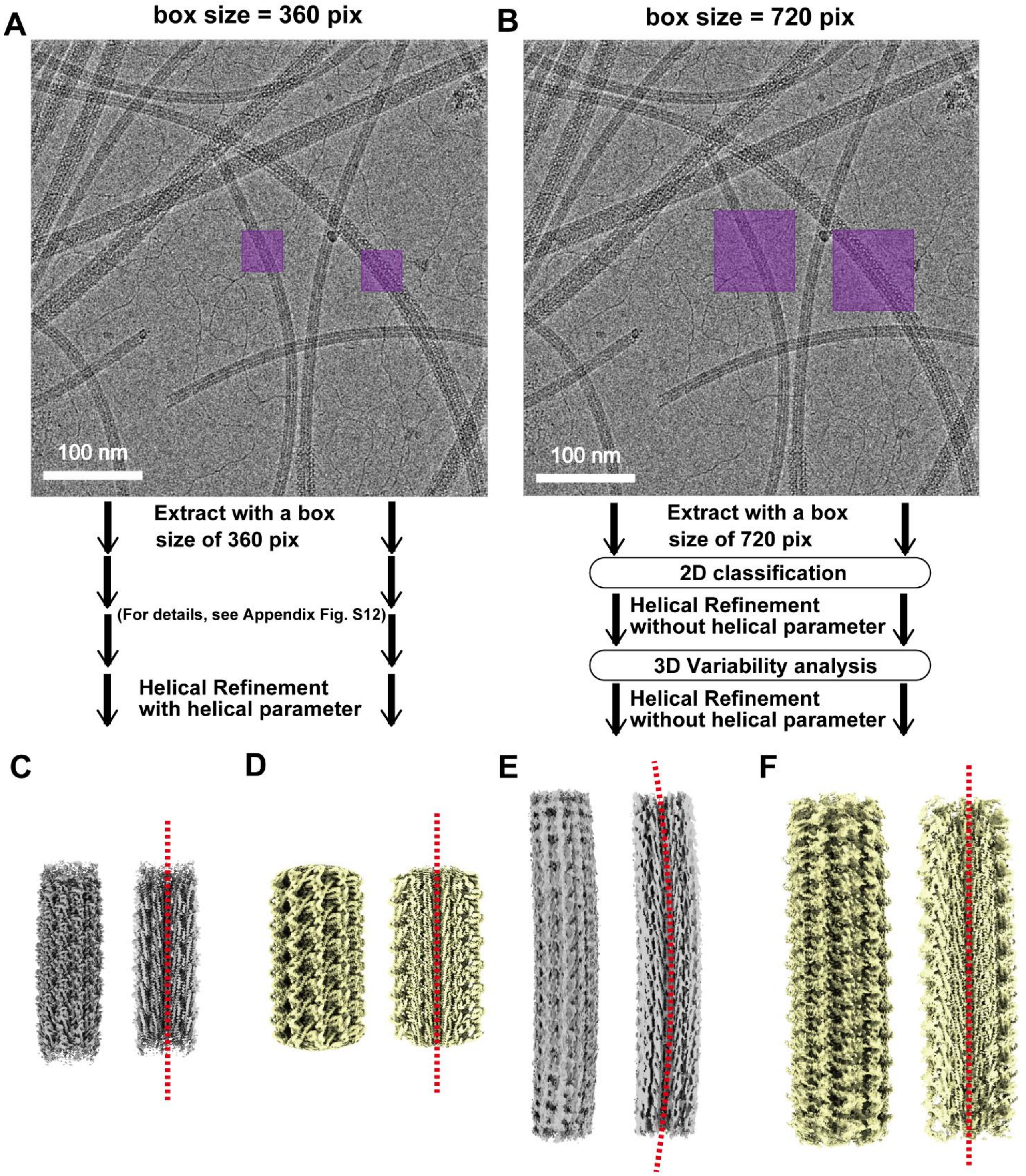

Appendix Fig. S14. Structural comparison of *ΔfcpB*_CL15 mutant PFs processed with and without applying helical symmetry.

A, B Data-processing workflows for helical refinement with (A) and without (B) applying helical symmetry. The areas from which particles were extracted are indicated by purple squares. For details, see Appendix Fig. S12.

C–F Reconstructed density maps and corresponding cross-sectional views of the core filament obtained with (C) and without (E) applying helical symmetry, and of the sheathed filament obtained with (D) and without (F) applying helical symmetry. Red dotted lines indicate the filament axis.
