# Supplementary figures and images for: Asymmetric sheath coordination controls flagellar architecture and function in Leptospira spirochete
Source: EMBO J. 2026 Mar 17;45(9):2882–904. doi: 10.1038/s44318-026-00731-1 (PMC13144727; doi:10.1038/s44318-026-00731-1)

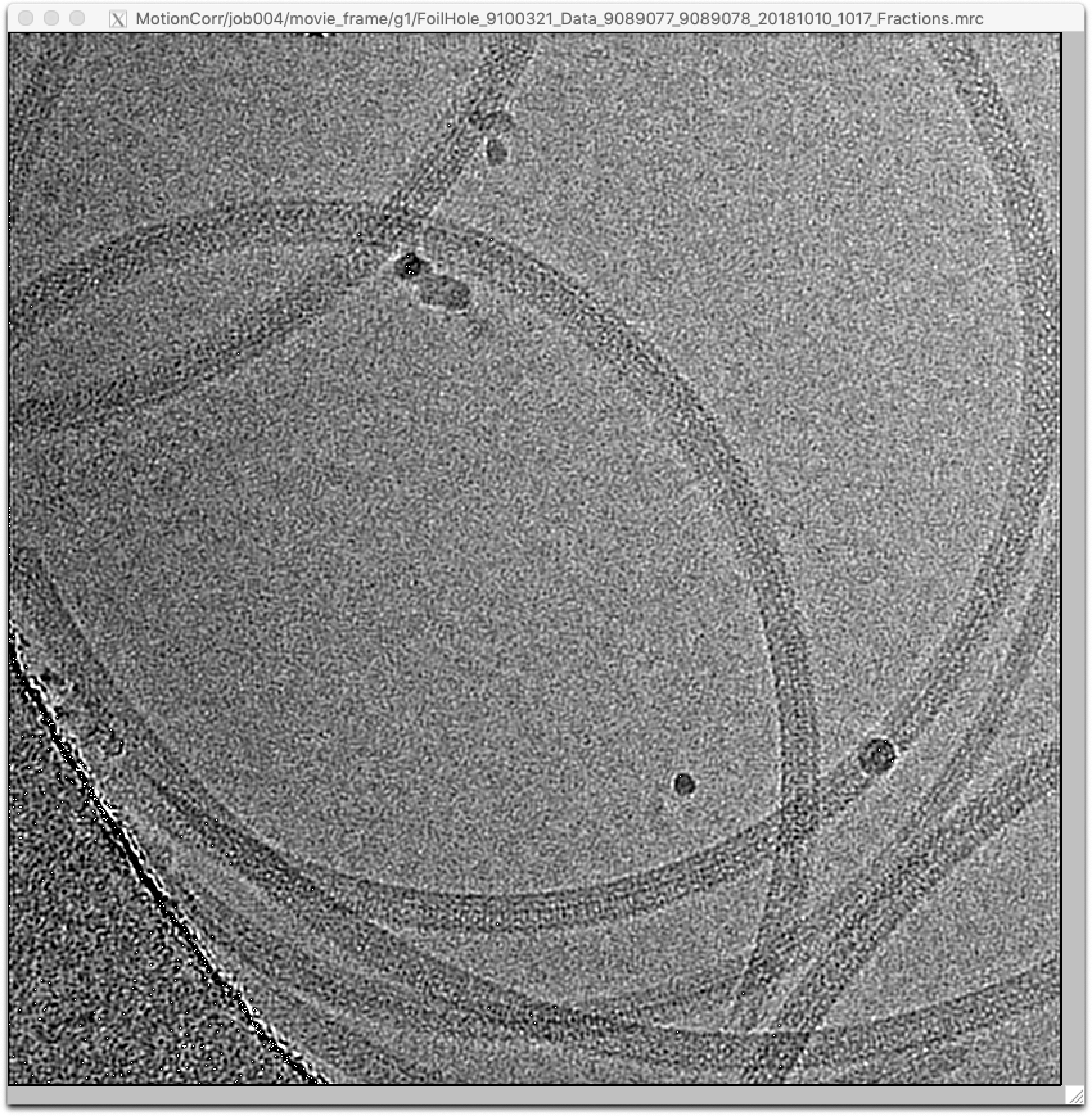

Supplement: Supplementary file 3 — Source data Fig. 1 [file 44318_2026_731_MOESM3_ESM.zip › Figure 1/1B/fig1B-original.png]

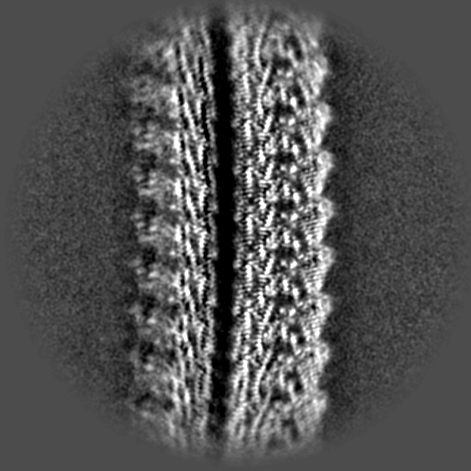

Supplement: Supplementary file 3 — Source data Fig. 1 [file 44318_2026_731_MOESM3_ESM.zip › Figure 1/1C/fig1C-original.tif]

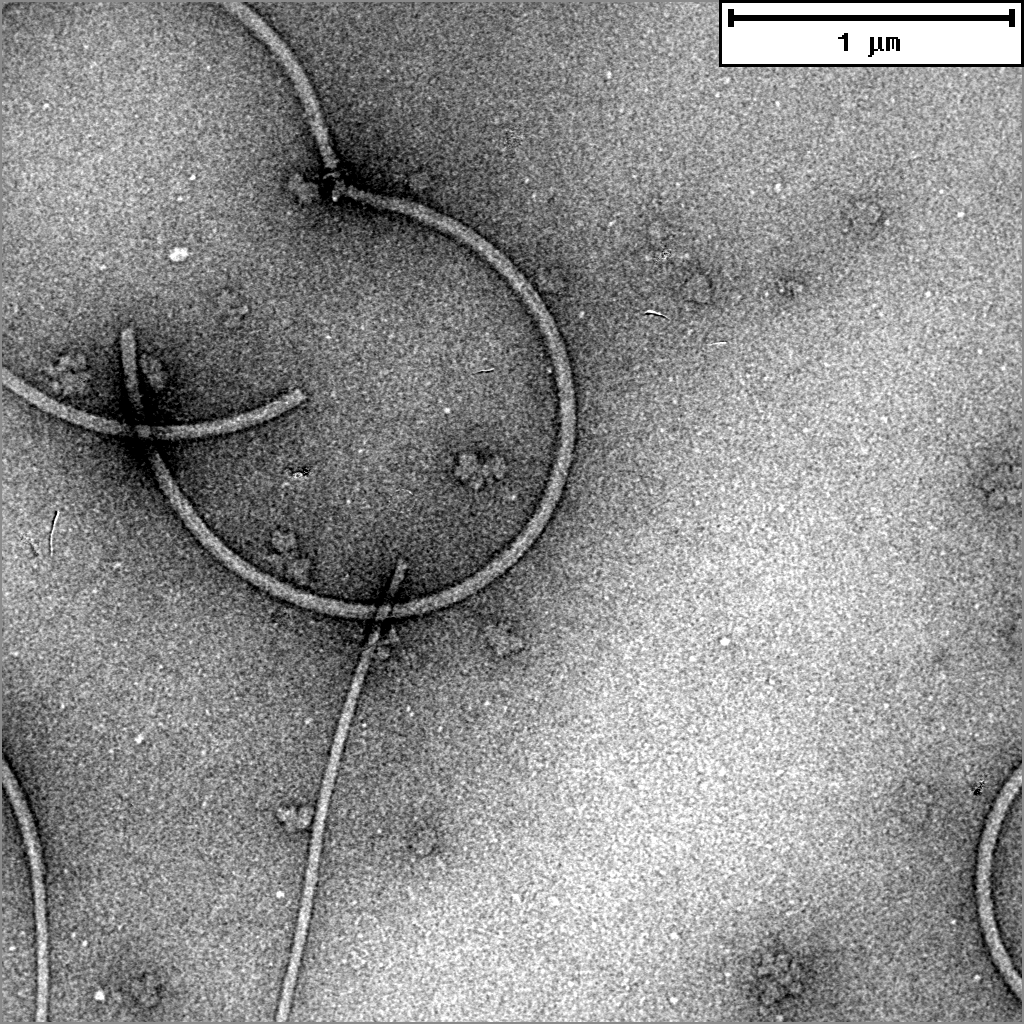

Supplement: Supplementary file 4 — Source data Fig. 2 [file 44318_2026_731_MOESM4_ESM.zip › Figure 2/2A/Lower_flaA2-complemented strain.tif]

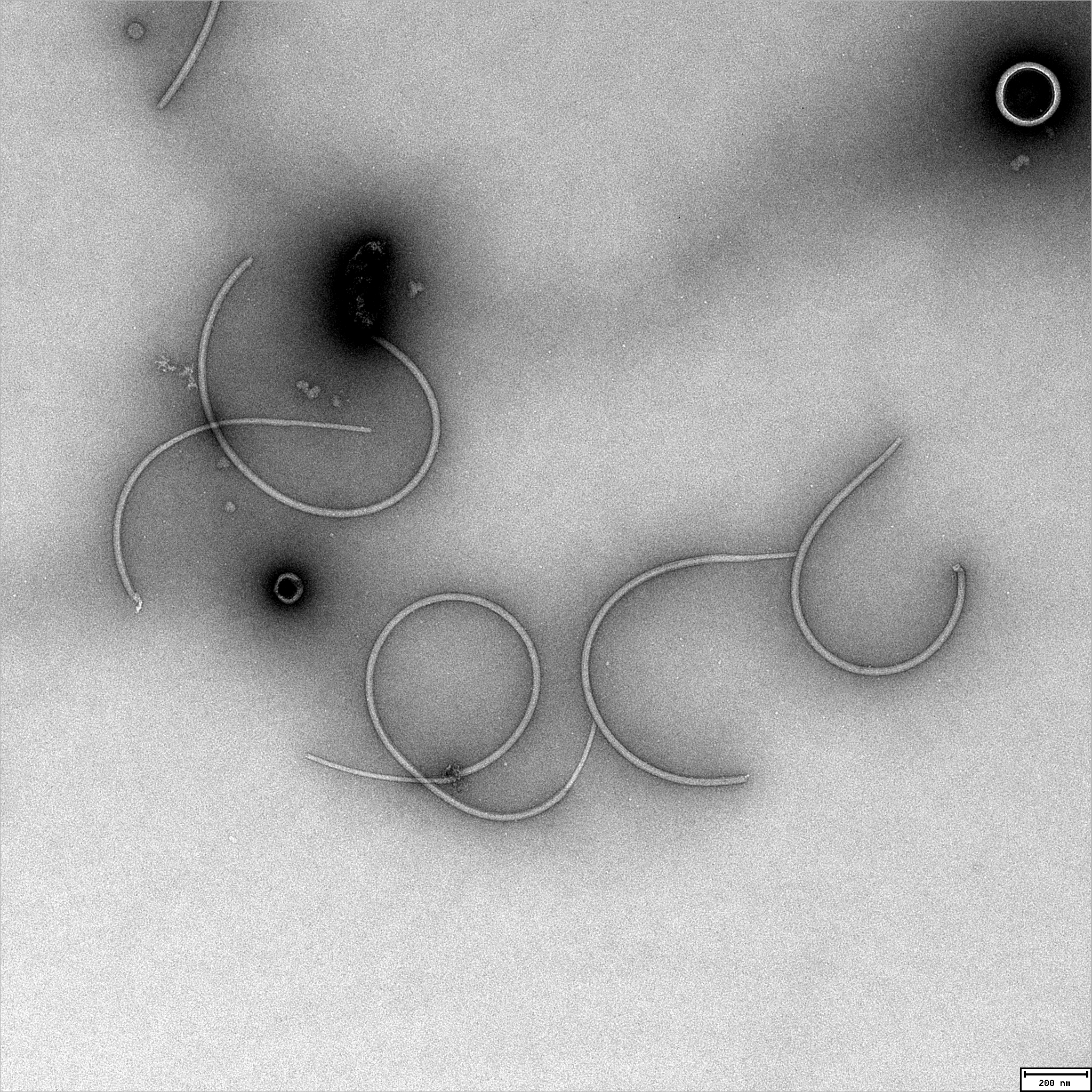

Supplement: Supplementary file 4 — Source data Fig. 2 [file 44318_2026_731_MOESM4_ESM.zip › Figure 2/2A/Lower_WT.tif]

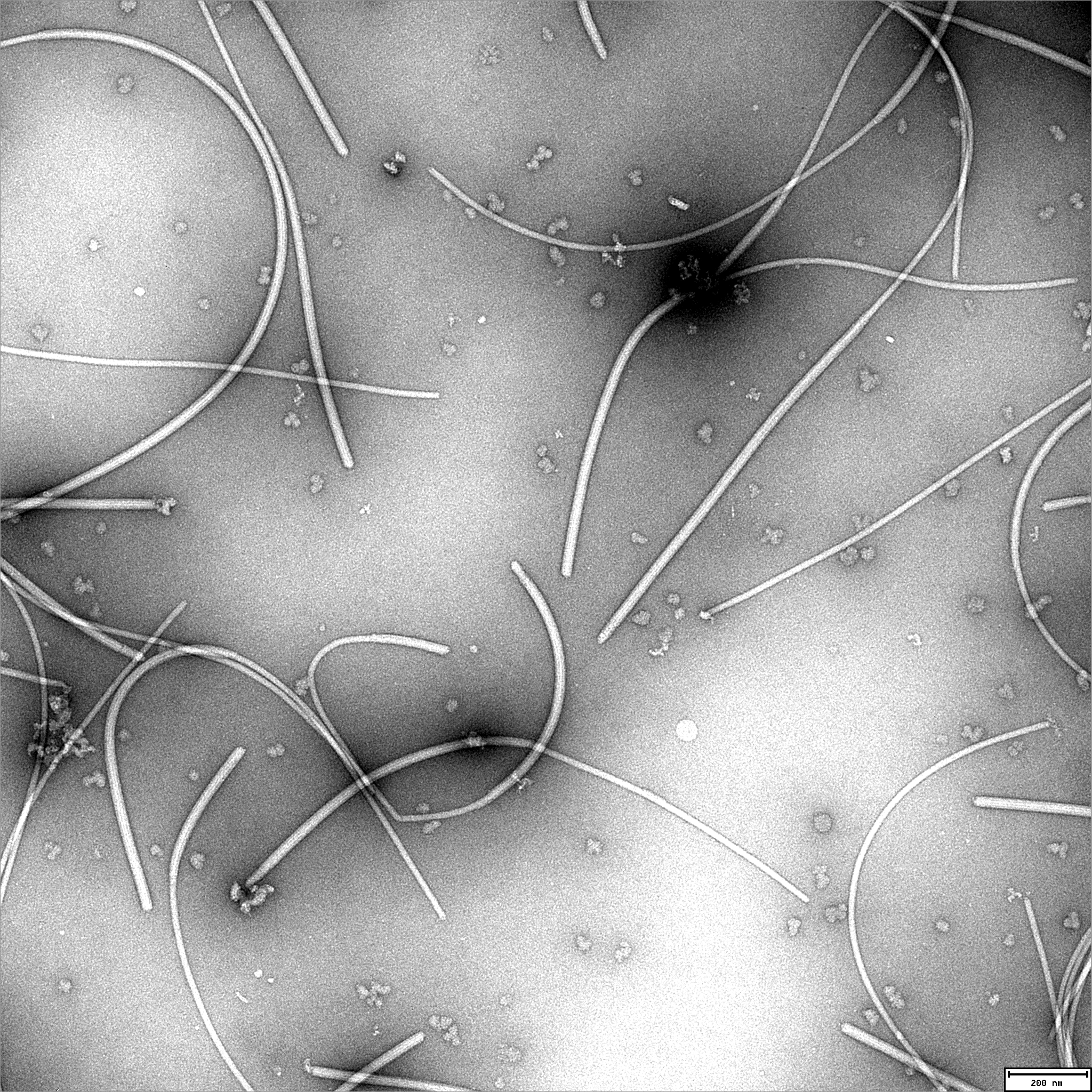

Supplement: Supplementary file 4 — Source data Fig. 2 [file 44318_2026_731_MOESM4_ESM.zip › Figure 2/2A/Lower_ΔflaA2.tif]

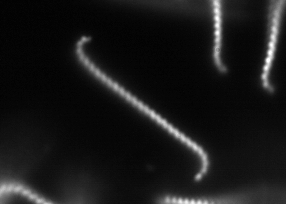

Supplement: Supplementary file 4 — Source data Fig. 2 [file 44318_2026_731_MOESM4_ESM.zip › Figure 2/2A/Middle_flaA2-complemented strain.png]

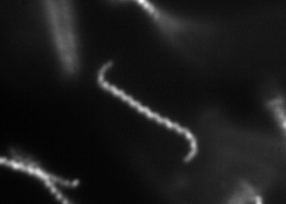

Supplement: Supplementary file 4 — Source data Fig. 2 [file 44318_2026_731_MOESM4_ESM.zip › Figure 2/2A/Middle_WT.png]

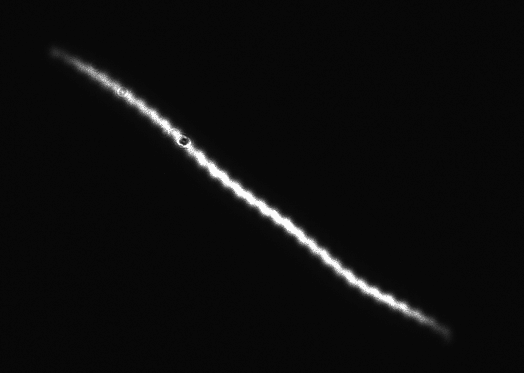

Supplement: Supplementary file 4 — Source data Fig. 2 [file 44318_2026_731_MOESM4_ESM.zip › Figure 2/2A/Middle_ΔflaA2.png]

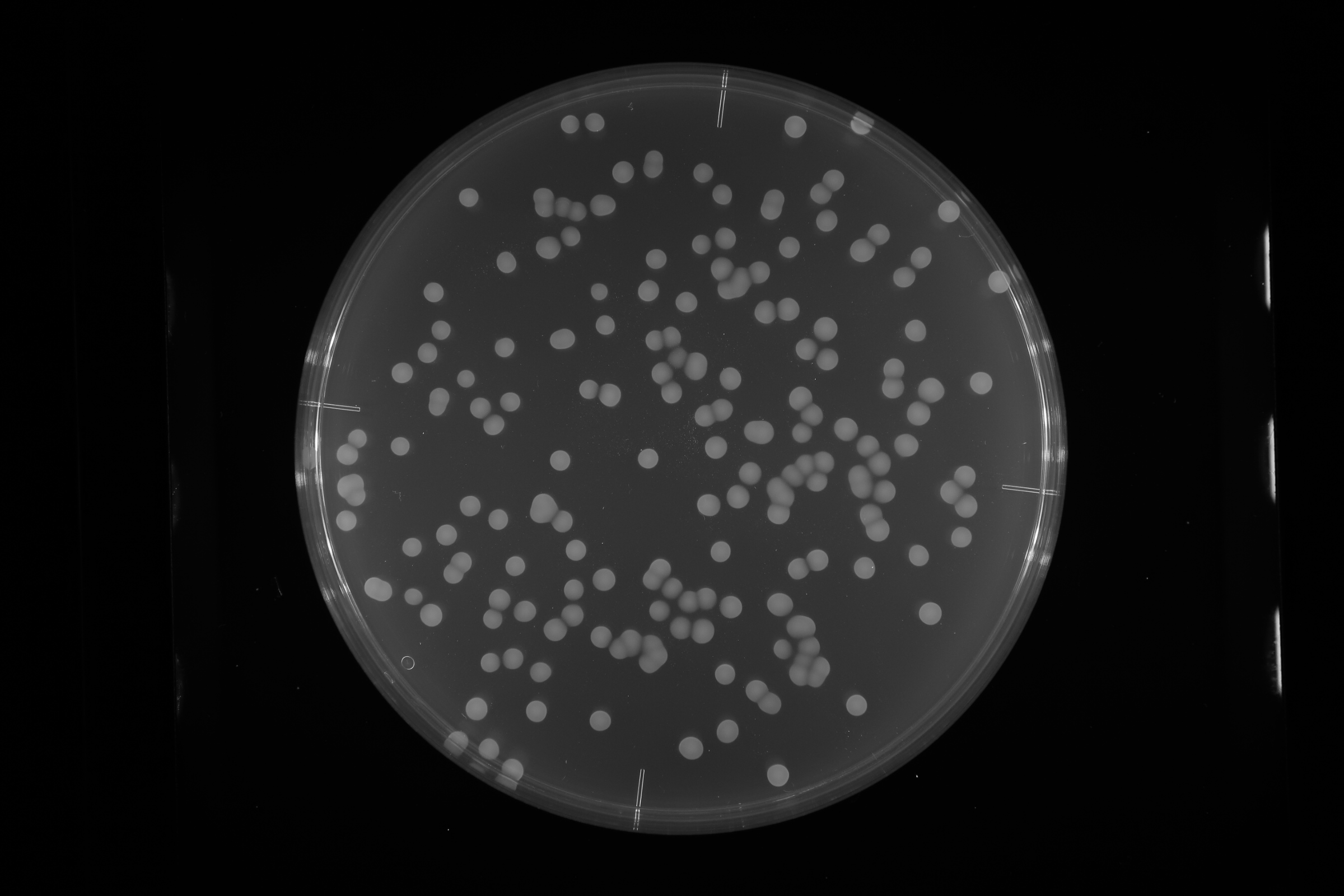

Supplement: Supplementary file 4 — Source data Fig. 2 [file 44318_2026_731_MOESM4_ESM.zip › Figure 2/2A/Upper_flaA2-complemented strain.JPG]

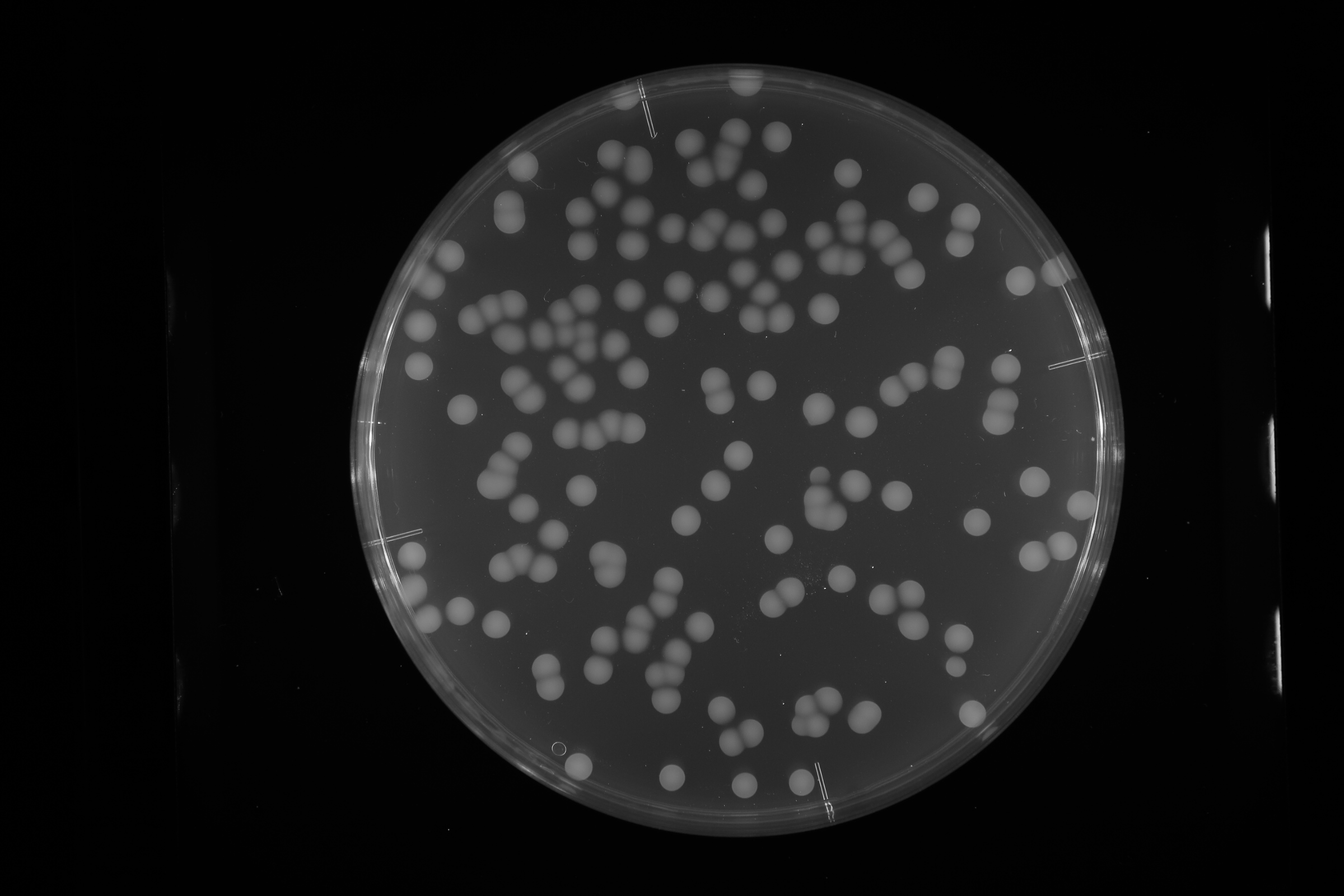

Supplement: Supplementary file 4 — Source data Fig. 2 [file 44318_2026_731_MOESM4_ESM.zip › Figure 2/2A/Upper_WT.JPG]

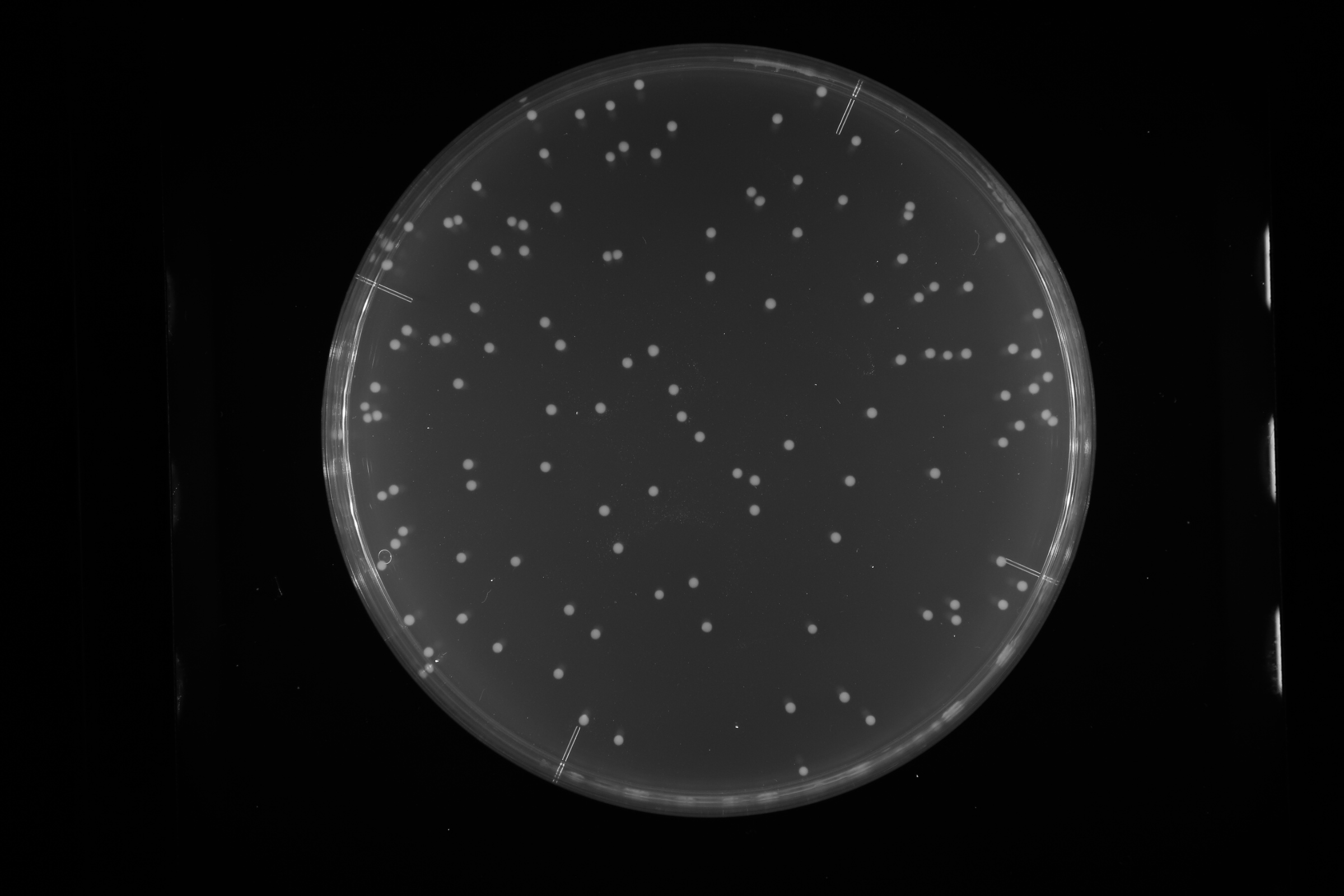

Supplement: Supplementary file 4 — Source data Fig. 2 [file 44318_2026_731_MOESM4_ESM.zip › Figure 2/2A/Upper_ΔflaA2.JPG]

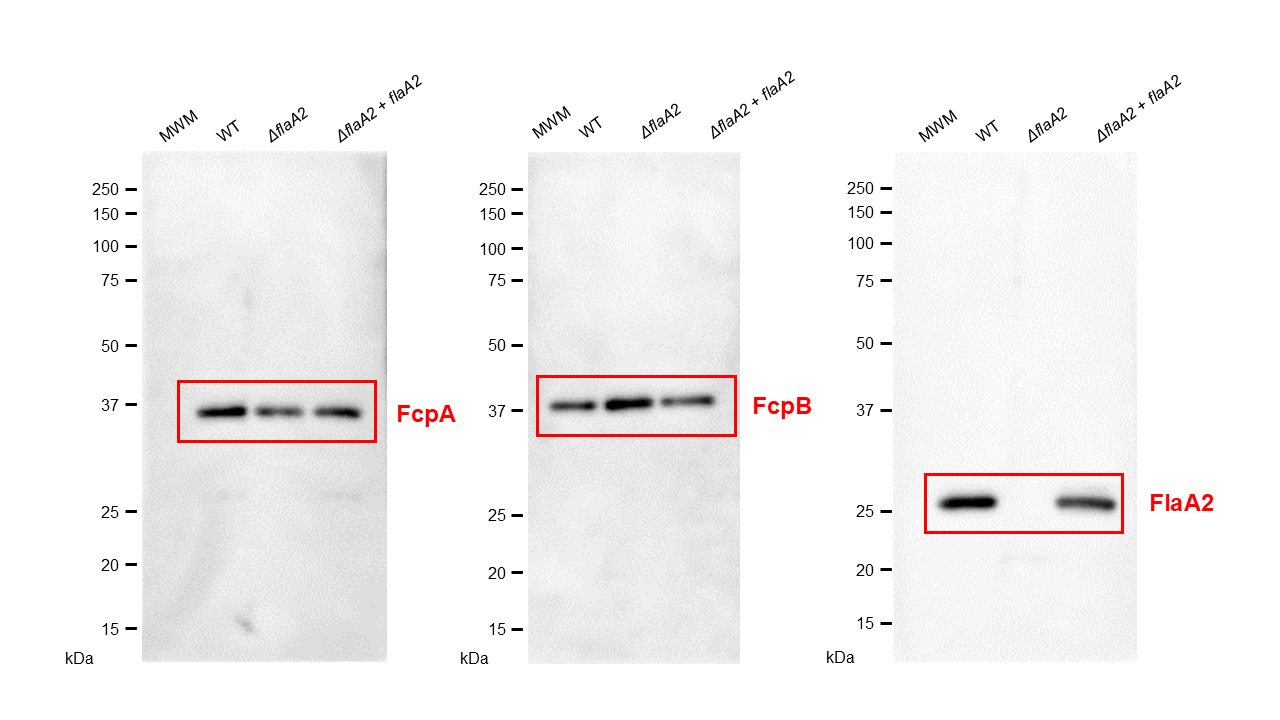

Supplement: Supplementary file 4 — Source data Fig. 2 [file 44318_2026_731_MOESM4_ESM.zip › Figure 2/2C/2C.TIF]

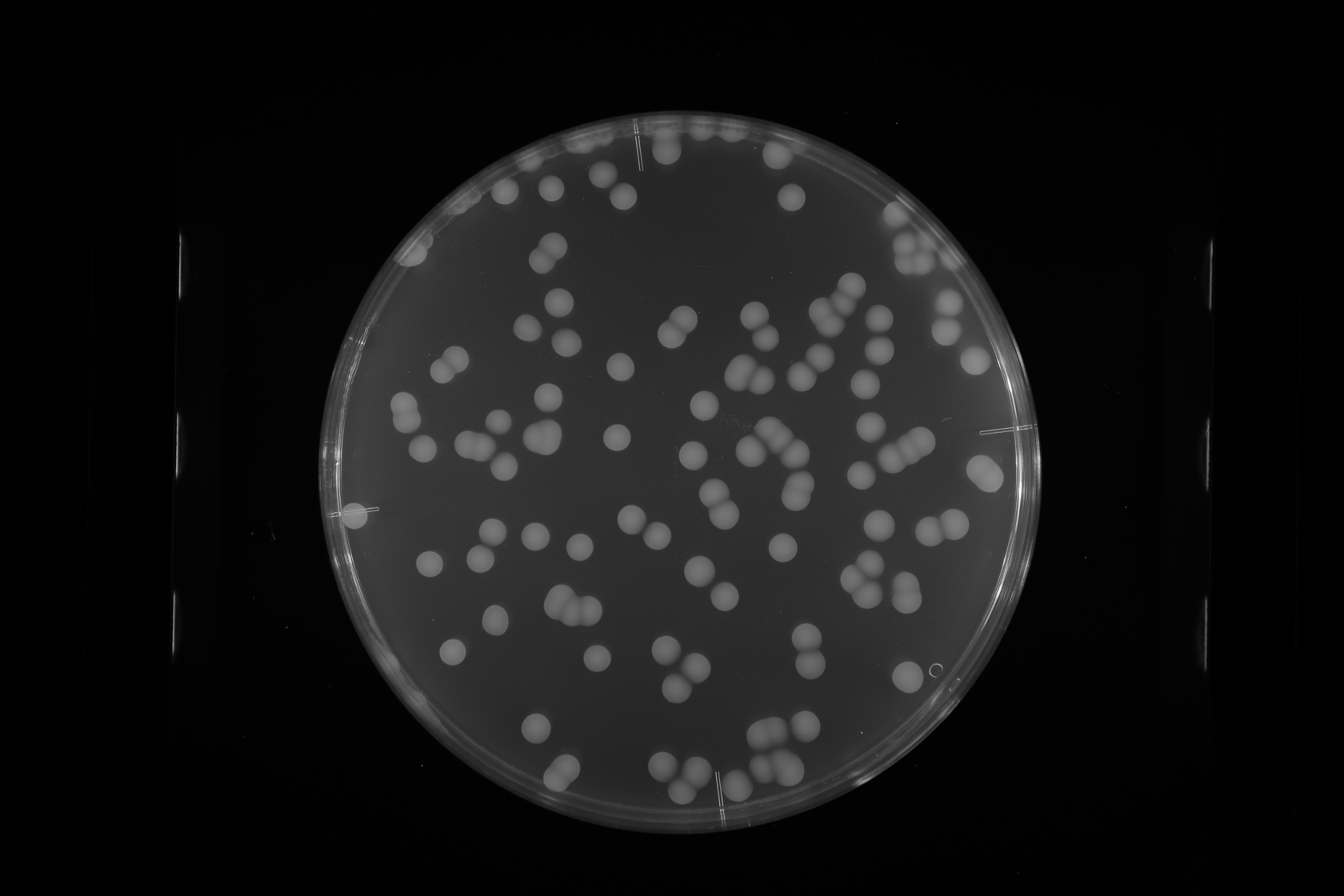

Supplement: Supplementary file 5 — Source data Fig. 4 [file 44318_2026_731_MOESM5_ESM.zip › Figure 4/4A/Wild type.JPG]

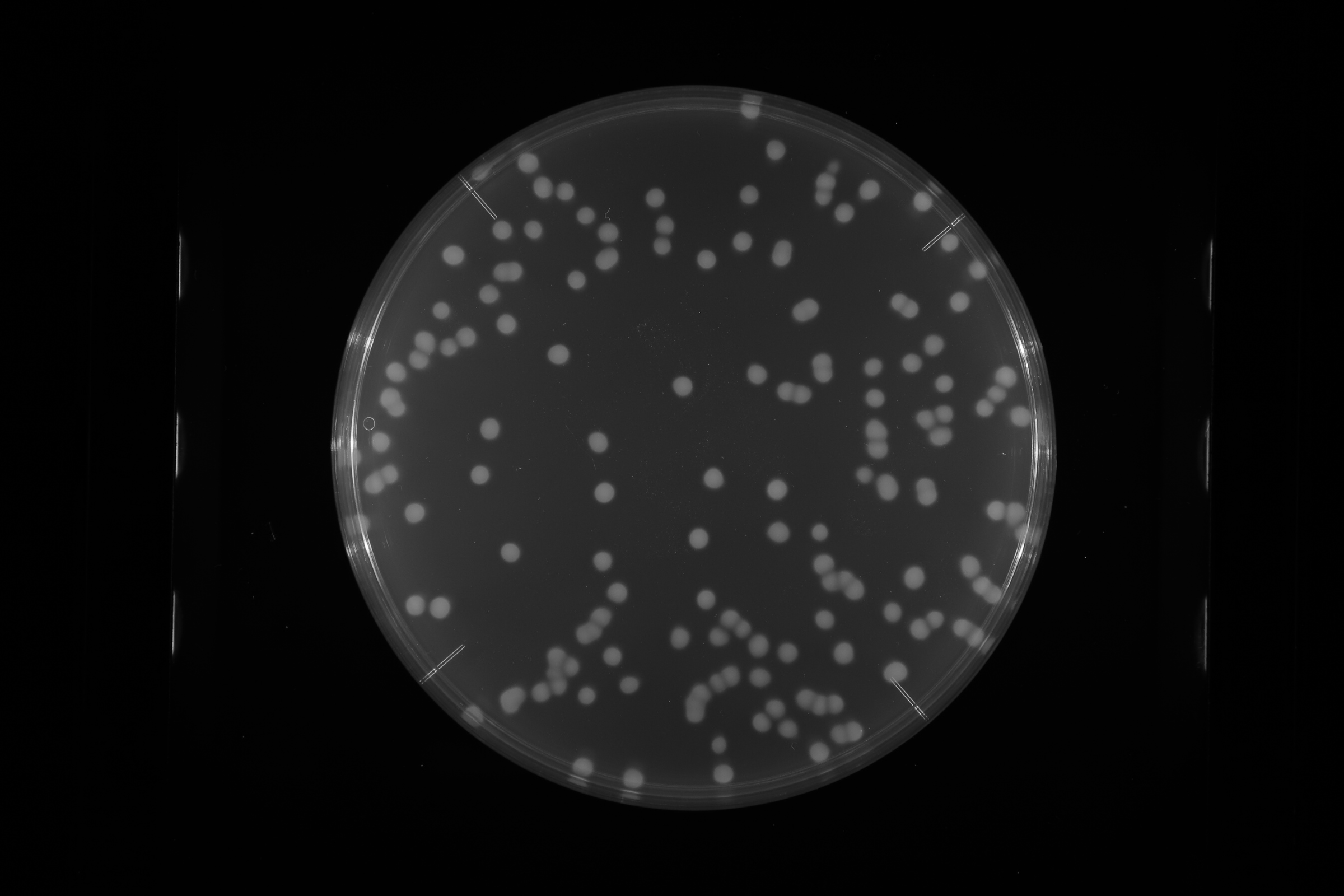

Supplement: Supplementary file 5 — Source data Fig. 4 [file 44318_2026_731_MOESM5_ESM.zip › Figure 4/4A/ΔfcpB_CL13.JPG]

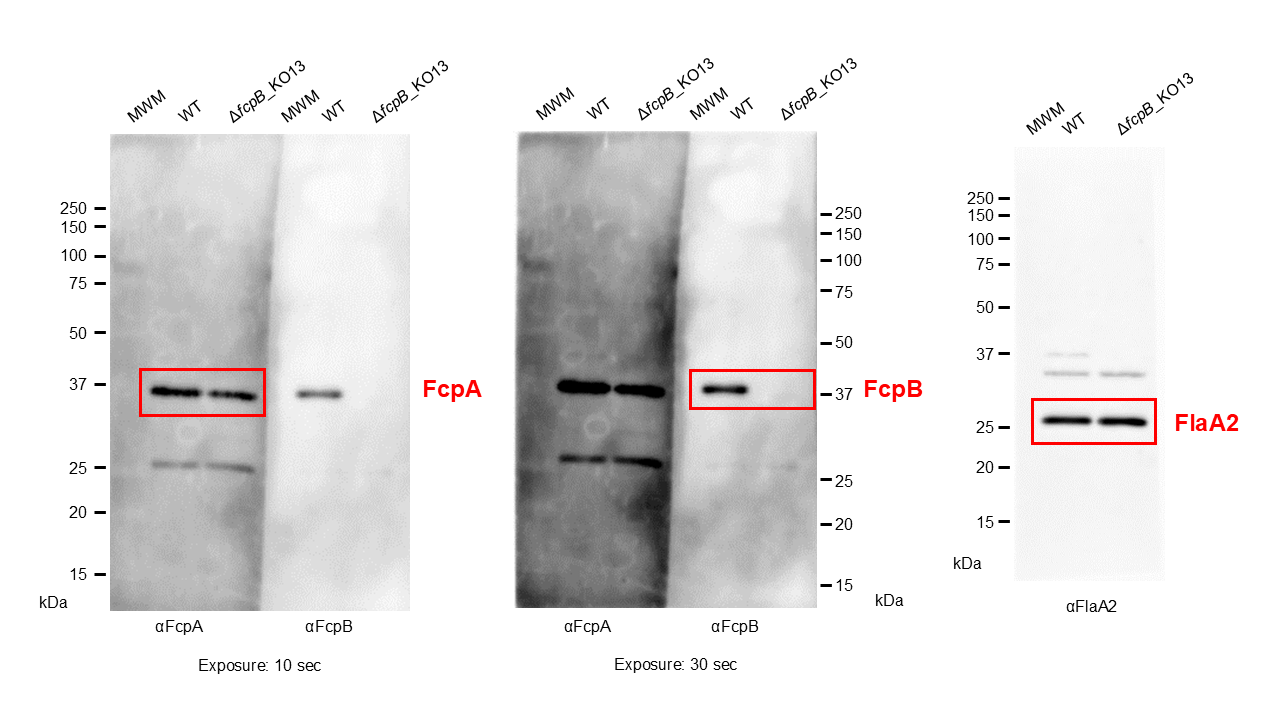

Supplement: Supplementary file 5 — Source data Fig. 4 [file 44318_2026_731_MOESM5_ESM.zip › Figure 4/4C/4C.TIF]

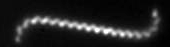

Supplement: Supplementary file 6 — Source data Fig. 5 [file 44318_2026_731_MOESM6_ESM.zip › Figure 5/5A/WT.bmp]

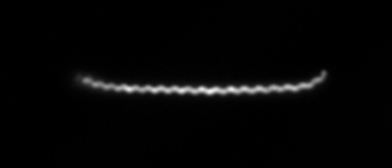

Supplement: Supplementary file 6 — Source data Fig. 5 [file 44318_2026_731_MOESM6_ESM.zip › Figure 5/5A/ΔfcpB.bmp]

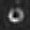

Supplement: Supplementary file 6 — Source data Fig. 5 [file 44318_2026_731_MOESM6_ESM.zip › Figure 5/5B/WT-1.bmp]

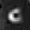

Supplement: Supplementary file 6 — Source data Fig. 5 [file 44318_2026_731_MOESM6_ESM.zip › Figure 5/5B/WT-2.bmp]

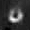

Supplement: Supplementary file 6 — Source data Fig. 5 [file 44318_2026_731_MOESM6_ESM.zip › Figure 5/5B/WT-5.bmp]

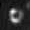

Supplement: Supplementary file 6 — Source data Fig. 5 [file 44318_2026_731_MOESM6_ESM.zip › Figure 5/5B/ΔfcpB-18.bmp]

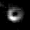

Supplement: Supplementary file 6 — Source data Fig. 5 [file 44318_2026_731_MOESM6_ESM.zip › Figure 5/5B/ΔfcpB-5.bmp]

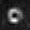

Supplement: Supplementary file 6 — Source data Fig. 5 [file 44318_2026_731_MOESM6_ESM.zip › Figure 5/5B/ΔfcpB-9.bmp]
