# Supplementary material for: Asymmetric sheath coordination controls flagellar architecture and function in Leptospira spirochete
Source: EMBO J. 2026 Mar 17;45(9):2882–904. doi: 10.1038/s44318-026-00731-1 (PMC13144727; doi:10.1038/s44318-026-00731-1)
Supplement: Supplementary file 7 — Expanded View Figures [file 44318_2026_731_MOESM7_ESM.pdf]

Expanded View Figures

Figure EV1. Summary of Cryo-EM data acquisition and image processing of periplasmic flagella (PFs) from wild-type strain.

(**A**) Cryo-EM image of purified PFs. Blue and red arrows indicate the unsheathed core filament and the sheathed filament, respectively. (**B**) Data-processing workflow. (**C**) Reconstructed image of the unsheathed core filament. (**D**) Reconstructed image of the sheathed filament. (**E**) 2D class image of the unsheathed core filament. (**F**) 2D class image of the sheathed filament. (**G**) Fourier shell correlation (FSC) analysis of the reconstructed unsheathed core filament, showing a global resolution of 4.35 Å. (**H**) FSC analysis of the reconstructed sheathed filament, showing a global resolution of 3.24 Å. (**I–L**) Fitted atomic models and corresponding density maps of FlaB2 (**I**), FlaB1 (**J**), FcpA (**K**), and FcpB (**L**).

▶

© The Author(s)

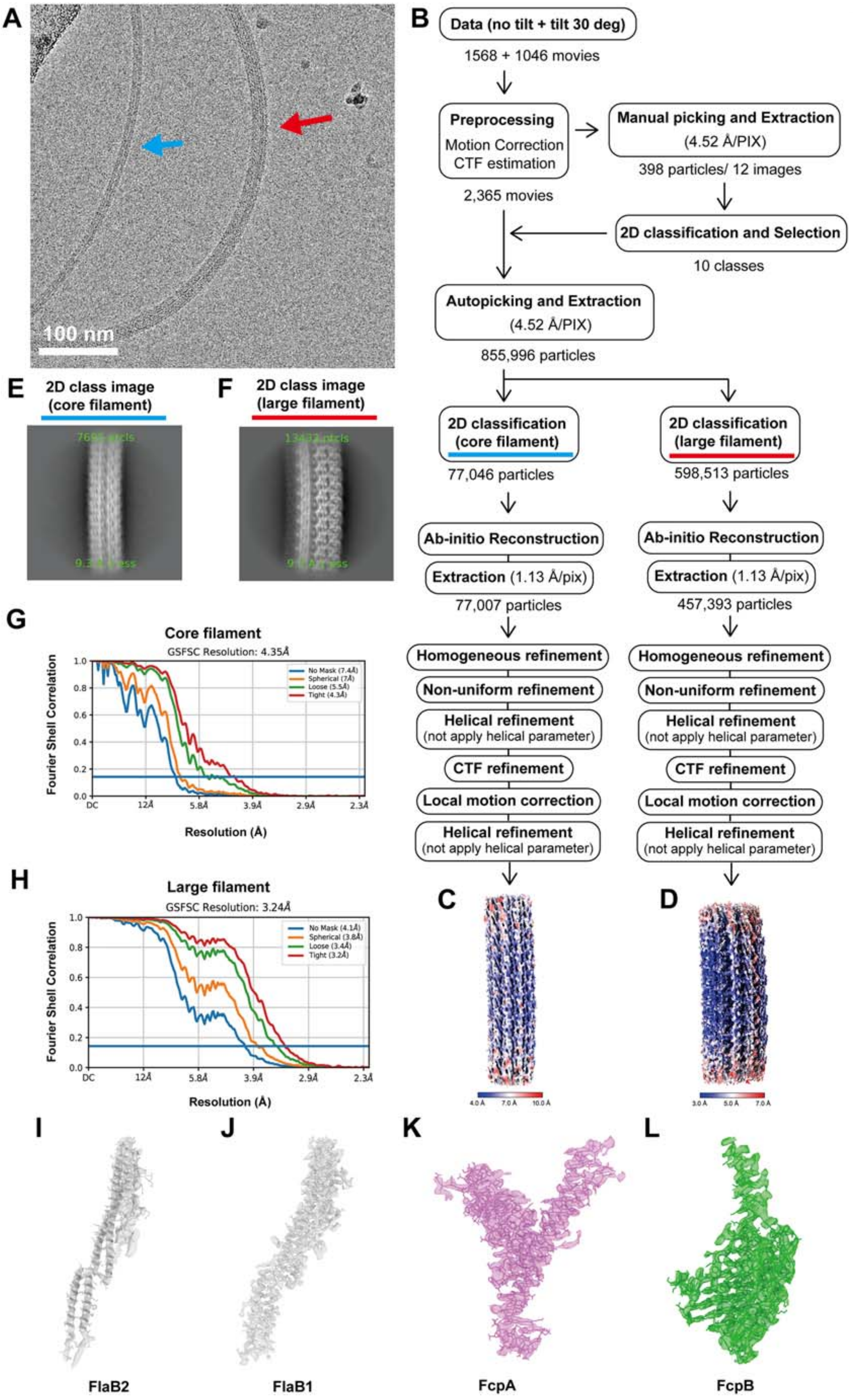
A
100 nm
B
Data (no tilt + tilt 30 deg)
1568 + 1046 movies
Preprocessing
Motion Correction
CTF estimation
2,365 movies
Manual picking and Extraction
(4.52 Å/PIX)
398 particles/ 12 images
2D classification and Selection
10 classes
Autopicking and Extraction
(4.52 Å/PIX)
855,996 particles
2D classification
(core filament)
77,046 particles
2D classification
(large filament)
598,513 particles
Ab-initio Reconstruction
Extraction (1.13 Å/pix)
77,007 particles
457,393 particles
Homogeneous refinement
Non-uniform refinement
Helical refinement
(not apply helical parameter)
CTF refinement
Local motion correction
C
D
E
2D class image
(core filament)
F
2D class image
(large filament)
G
Core filament
GSFSC Resolution: 4.35Å
No Mask (7.4Å)
Spherical (7Å)
Loose (5.5Å)
Tight (4.3Å)
Fourier Shell Correlation
Resolution (Å)
H
Large filament
GSFSC Resolution: 3.24Å
No Mask (4.1Å)
Spherical (3.8Å)
Loose (3.4Å)
Tight (3.2Å)
I
J
K
L
FlaB2
FlaB1
FcpA
FcpB

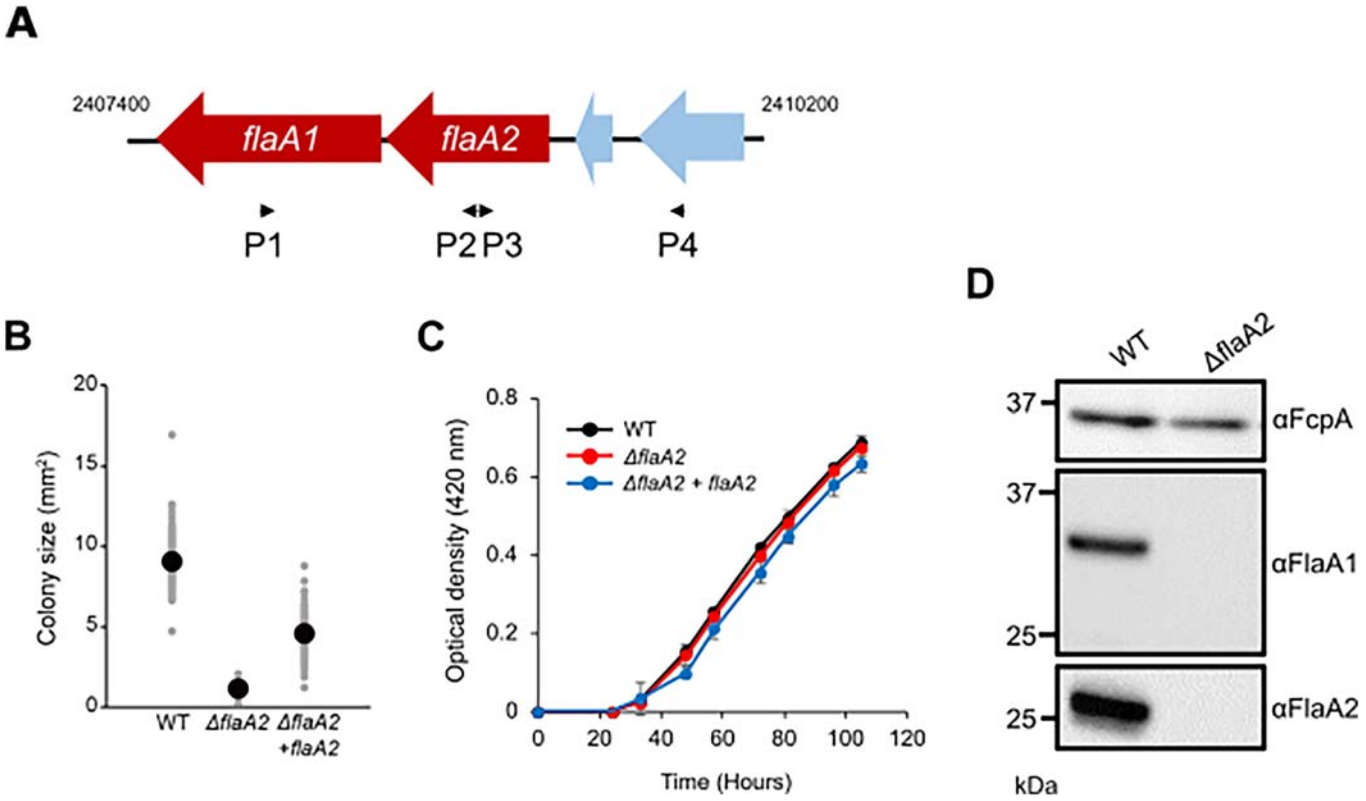

Figure EV2. ***ΔflaA2*** **mutant expresses neither FlaA2 nor FlaA1.**

(**A**) Schematic diagram of the genomic region (positions 2407400 to 2410200) of *L. biflexa* serovar Patoc strain Patoc I (Paris) (GenBank accession no. CP000786.1), showing the relative positions and orientations of *flaA2* and *flaA1*. The locations of primers used for constructing the *flaA2* deletion mutant (P1: 2407983–2408003, P2: 2408872–2408895, P3: 2408896–2408916, P4: 2409784–2409803) are indicated by black arrows. (**B**) Quantification of colony size of wild-type (WT), *ΔflaA2*, and *flaA2*-complemented (*ΔflaA2* + *flaA2*) strains. strains. All individual data points are plotted, with mean values indicated by black circles. Data were collected from 131 colonies for WT, 194 colonies for *ΔflaA2*, and 176 colonies for *flaA2*-complemented srains, obtained from two independent biological replicates. Statistical significance was assessed using a two-tailed Student's *t*-test, assuming unequal variances (WT vs *ΔflaA2* $P = 6.94 \times 10^{-96}$, WT vs *ΔflaA2* + *flaA2* $P = 8.29 \times 10^{-75}$, *ΔflaA2* vs *ΔflaA2* + *flaA2* $P = 2.50 \times 10^{-94}$). (**C**) Growth of *L. biflexa* WT (black), *ΔflaA2* (red), and *flaA2*-complemented (blue) strains. Optical density (OD) at 420 nm was measured at 0, 24, 33, 48, 51, 72, 81, 96, and 105 h after inoculation. Measurements were terminated at 105 h (day 4), as cell sedimentation occurred after day 5. For each independent experiment, OD measurements were performed in triplicate, and the triplicate values were averaged to obtain a single data point. Data points represent the mean of three independent experiments, and error bars indicate the standard deviation. The culture medium for the *flaA2*-complemented strain contained 25 μg/mL kanamycin. (**D**) Immunoblotting of whole-cell lysates from WT and *ΔflaA2* mutant using anti-FcpA, anti-FlaA1, and anti-FlaA2 antisera. Whole-cell lysates were prepared in two independent experiments, and each preparation was analyzed by immunoblotting; a representative blot is shown.

 © The Author(s)

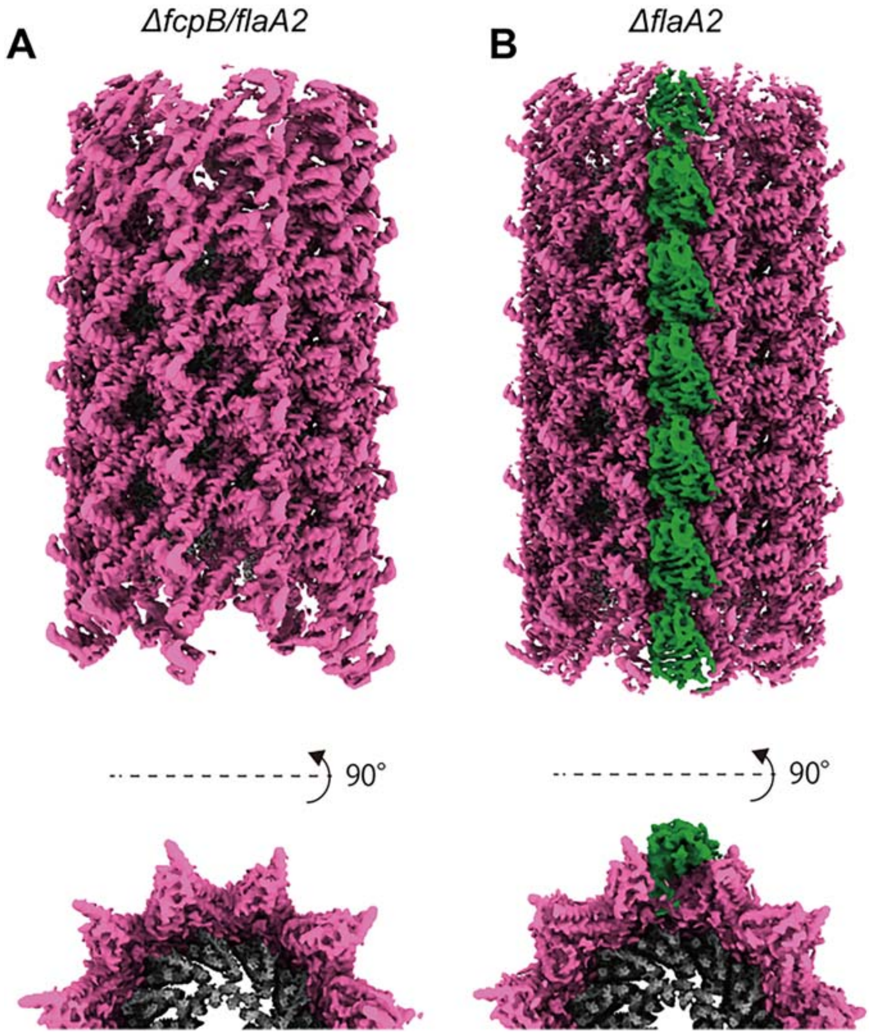

Figure EV3. Interaction of FcpB with FcpA.

(**A**) Reconstructed image of the periplasmic flagellum (PF) from *ΔfcpB/flaA2* mutant, which is uniformly covered by FcpA alone. The FlaB core filament (gray) and FcpA (magenta) are shown. (**B**) Reconstructed image of the PF from *ΔflaA2* mutant, uniformly covered by both FcpA and FcpB. FlaB (gray), FcpA (magenta), and FcpB (green) are shown. See Methods for details of reconstruction reproducibility.

© The Author(s)

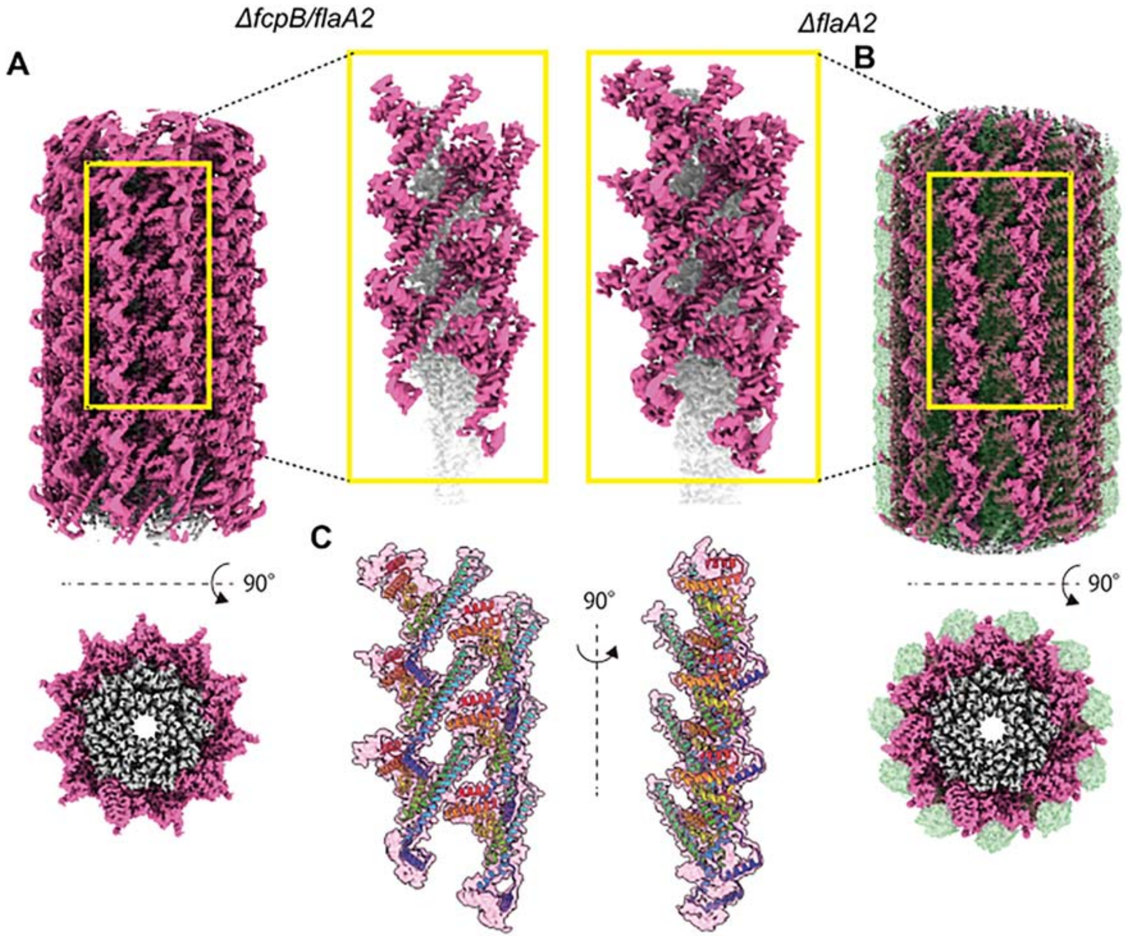

Figure EV4. Effect of FcpB on the structure of FcpA row.

(**A**) Reconstructed image of the periplasmic flagellum (PF) from *ΔfcpB/flaA2* mutant, which is uniformly covered by FcpA alone. The FlaB core filament (gray) and FcpA (magenta) are shown. A magnified view of the boxed region is shown on the right. (**B**) Reconstructed image of the PF from *ΔflaA2* mutant, uniformly covered by both FcpA and FcpB. FlaB (gray), FcpA (magenta), and FcpB (green) are shown. In the magnified view (left), FcpB is omitted to highlight FcpA structure. (**C**) Structural comparison of FcpA between the two mutants. The FcpA model from *ΔfcpB/flaA2* mutant (shown as a rainbow-colored ribbon, from panel A) is superimposed onto the FcpA density map from *ΔflaA2* mutant (magenta, from panel **B**). See Methods for details of reconstruction reproducibility.

 © The Author(s)

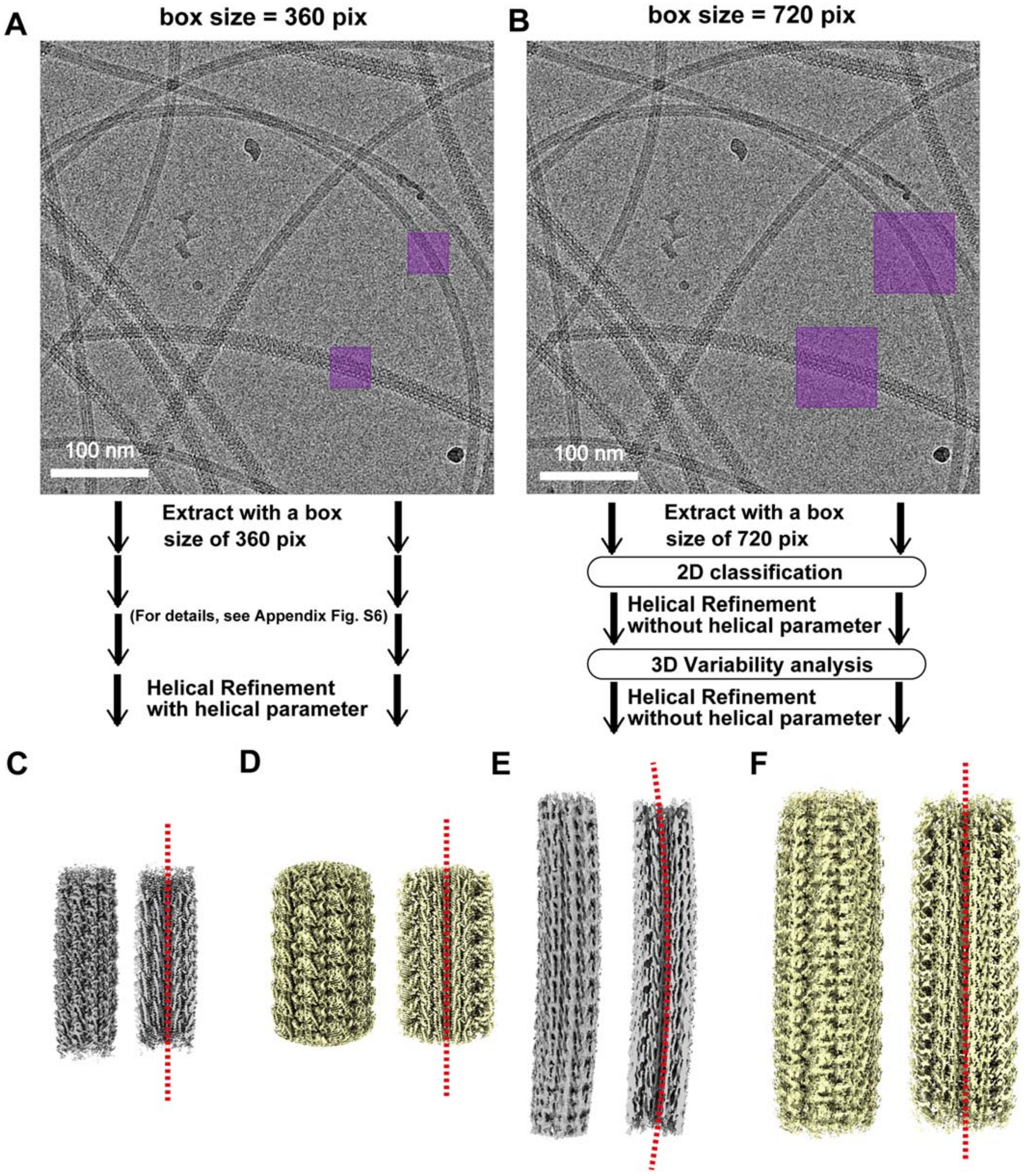

Figure EV5. Structural comparison of Δ*flaA2* mutant PFs processed with and without applying helical symmetry.

(**A**, **B**) Data-processing workflows for helical refinement with (**A**) and without (**B**) applying helical symmetry. The areas from which particles were extracted are indicated by purple squares. For details, see Appendix Fig. S6. (**C–F**) Reconstructed density maps and corresponding cross-sectional views of the core filament obtained with (**C**) and without (**E**) applying helical symmetry, and of the sheathed filament obtained with (**D**) and without (**F**) applying helical symmetry. Red dotted lines indicate the filament axis.

© The Author(s)
